# Towards greater sensitivity: advancing social life cycle assessment for the coffee sector in Brazil and the Democratic Republic of the Congo

**DOI:** 10.1007/s11367-026-02760-8

**Published:** 2026-10-18

**Authors:** Braida Thom, Lea Fabritius, Marie Sigrist, Didace Banyanga, Stephan Pfister, Réussite Bugale Malembaka, Gabriela Schneider, Kokou Kintche, Johanna Jacobi

**Affiliations:** 1 Agroecological Transitions, Department of Environmental Systems Sciences, ETH Zurich, Universitätstrasse 2, Zurich, 8092 Switzerland; 2 International Institute of Tropical Agriculture (IITA), Route Kavumu, Km 18, Bifurcation Birava, Site Kalambo, Bukavu, Democratic Republic of Congo; 3 Ecological Systems Design, Department of Civil, Environmental and Geomatic Engineering, ETH Zurich, Laura-Hezner-Weg 7, Zurich, 8093 Switzerland; 4 Programa de Ciencias Sociais em Desenvolvimento, Agricultura e Sociedade, Universidade Federal Rural do Rio de Janeiro (UFRRJ), BR-465, Km 7 in Seropédica Rio de Janeiro, Brazil

**Keywords:** Social performance, Primary data, Local contextualization, Reference scale approach, Participation, Coffee value chain

## Abstract

**Purpose:**

Literature on coffee sustainability focuses on environmental and economic challenges and often falls short on social factors that strongly influence overall sustainability. For coffee producers, social aspects are existentially important, while environmental concerns take lower priority. Recognizing that social aspects are critical to sustainable development, we assessed the social impacts of nine coffee case studies in Brazil and the Democratic Republic of the Congo (DRC).

**Methods:**

Social Life Cycle Assessment (S-LCA) has gained interest and importance to better understand and measure the positive and negative social impacts of products on various stakeholders. However, many challenges in its applicability still exist. Therefore, in this study, we operationalized and applied the S-LCA methodology for the coffee sector, based on the UNEP guidelines and the ISO standard 14,075. We used the reference scale approach based only on primary, site-specific data and systematically involved different actors at the upstream stages of the coffee value chain to capture multiple perspectives. In total, we conducted 170 inventory interviews and included six validation interviews.

**Results:**

We found significant differences in social performance both within and across the two countries; cross-country differences are reported to contrast the two contexts rather than to rank performance. The differences we found within the two countries show that not only context but also organization matters. Especially in Brazil, the cooperative models scored higher than the commercial plantations. In the Brazilian case studies, mean performance scores were highest for the stakeholder category ‘farmer’, followed by ‘local community’, ‘worker’, and ‘value chain actors’. In the Congolese case studies, mean performance scores were highest for the stakeholder category ‘local community’, followed by ‘farmer’, ‘worker’, and ‘value chain actors’. The impact subcategories showing the lowest social performance scores, such as ‘fair salary’, ‘livelihood and well-being’, and ‘productivity’, are the ones that were ranked highest during the validation interviews. We found that workers mostly do not earn living wages, with only seven exceptions in Brazil and one in the DRC.

**Conclusions:**

This study contributes to the limited body of S-LCA research on coffee production, and, to our knowledge, is among the first to be based on primary data collected in Brazil and the DRC. It presents a novel approach for achieving greater case sensitivity in S-LCA through primary data and context-specific reference scales, going beyond generic databases. This approach makes it possible to account for differences among case studies within the same country and sector.

**Supplementary Information:**

The online version contains supplementary material available at https://doi.org/10.1007/s11367-026-02760-8.

**Table 1:** Description of case studies and sample size

Case study	B1: direct trade cooperative	B2: women’s group	B3: social movement	B4: alternative specialty coffee plantation	B5: conventional specialty coffee plantation	D1: metayage^f^ plantation		D2: female-led cooperative	D3: established cooperative	D4: emerging cooperative
**Location**	South of Minas Gerais, Brazil					South Kivu, DRC				
**Characteristics** (from: Thom et al. 2025, p.4)	“Small, innovative, Fairtrade and partly organic certified cooperative with a focus on direct trade”	“Women’s group of an innovative, Fairtrade and partly organic certified cooperative with a focus on family farming”	“Cooperative of a social movement^d^ struggling for land redistribution and partly organic certified”	“Medium-sized, alternative commercial specialty coffee plantation, partly organic, partly conventional”	“Medium-sized, conventional commercial specialty coffee plantation”	“Large commercial coffee plantation linked to a multinational buyer and working with the *metayage* system”	“Female-led family farming cooperative linked to a multinational buyer and entering the specialty coffee export market”		“Established family farming cooperative exporting specialty coffee, partly directly to roasters”	“Emerging family farming cooperative entering the specialty coffee export market”
**Members (n)**	70 (16 female, 54 male)	504 (87 female, 417 male), 30 participate in women’s group	47 (18 female, 29 male)	-	-	-	1444 (953 female, 491 male)		2388 (422 female, 1966 male)	1245 (340 female, 905 male)
**Employees (n)**	7 permanent (3 female, 4 male)	68 permanent (31 female, 37 male)	8 permanent (3 female, 5 male)	19 permanent (10 female, 9 male), varying number of seasonal workers^e^	30 permanent (5 female, 25 male), varying number of seasonal workers	24 permanent (3 female, 21 male), 375 *metayers*^g^ (249 female, 126 male), varying number of day laborers	11 permanent (5 female, 6 male), 16 seasonal workers, varying number of day laborers		47 permanent (10 female, 37 male), 65 seasonal workers, varying number of day laborers	5 permanent (1 female, 4 male), 24 seasonal workers, varying number of day laborers
**Certifications**	Fairtrade (*n* = 9), third party organic certification (*n* = 5), Certifica Minas (*n* = 6), Rainforest Alliance (*n* = 1), Participatory Guarantee Systems (*n* = 1)	Fairtrade (*n* = 9), Participatory Guarantee Systems (*n* = 9), third party organic certification (*n* = 4)	third party organic certification (*n* = 3), Participatory Guarantee Systems (*n* = 3), Fairtrade (*n* = 2)	third part organic certification, Certifica Minas	Rainforest Alliance, Certifica Minas	third party organic certification		third party organic certification (*n* = 5)	Symbole Producteurs Paysans (*n* = 10)	-
**Post-harvest processing**	natural and honey	natural	natural	natural, honey, and fully-washed	natural, honey, and fully-washed	fully-washed		fully-washed	fully-washed	fully-washed
**Sample size (n)**	9 workers^a^, 10 farmers^b^, 1 management^c^	10 workers, 10 farmers, 1 management	6 workers, 10 farmers, 1 management	10 workers, 1 management (owner)	11 workers, 1 management (owner)	9 workers, 10 *metayers*, 5 management		8 workers, 10 farmers, 3 management	9 workers, 10 farmers, 4 management	8 workers, 10 farmers, 3 management

^a^**Workers**: Rural/farm workers, day laborers, technicians and office staff employed by a coffee plantation or cooperative. More information on their functions is provided in Table SI 1

^b^**Farmers**: Small- to medium-sized family farmers who either do all the work themselves or employ few people, usually on a temporary basis

^c^**Management**: Decision makers: People who own medium to large plantations or people who manage cooperatives or companies. More information on their functions is provided in Table SI 2

^d^**The social movement** is struggling for the implementation of the agrarian land reform and for social justice through the occupation of uncultivated land from large landowners

^e^**Seasonal workers** are mostly employed during the coffee harvest on coffee plantations (in Brazil) or in coffee washing stations (in the DRC). In Brazil, they are called ‘safristas’

^f^**The*metayage*system** is a form of sharecropping system where *metayers* are allowed to grow crops on the plantation’s land in exchange for 1–2 days of free labor per week

^g^***Metayers***: A type of sharecropper who are allowed to grow crops on someone else’s land in exchange for 1–2 days of free labor per week

**Table 2:** Reference scale with levels from − 1 to 2

Reference Scale			
-1	0	1	2
Non-compliant situation	Compliance with local laws/basic societal expectations/common practices in the sector	Proactive efforts/ progress beyond compliance	Evidence of outstanding efforts/positive output achieved/ideal performance
Example: **‘Lowest paid worker**,** compared to the minimum wage and living wage’** (stakeholder category ‘worker’)			
Lowest paid workers earn wages that are below the legal minimum wage and/or fall below the poverty line	Lowest paid workers earn the legal minimum wage	Lowest paid workers earn wages that are above the legal minimum wage but still below the living wage	Lowest paid workers earn wages that meet or exceed the minimum living wage

## Introduction

Literature on coffee sustainability focuses mainly on environmental and economic challenges and often neglects social factors, which strongly influence (environmental) sustainability (Bray et al. 2002; Brenes-Peralta et al. 2021; Rehman et al. 2025). For coffee producers, however, social aspects, such as meeting basic needs (e.g. food and decent housing) or the benefits of being organized, are often considered as most important, while environmental aspects take lower priority (Bray et al. 2002; Thom et al. 2025). Bray et al. (2002) even argue that changing the social matrix and empowering smallholder farmers is a prerequisite for transitioning to more ecological practices. Social aspects are therefore critical to achieving genuine sustainable development (Sivaraman 2020; Luna Ostos et al. 2024).

In most coffee origin countries, the average income from coffee cultivation is not sufficient to support farmers’ livelihoods. Moreover, since the Covid-19 pandemic, the share of farmers living below the poverty line has increased dramatically (Panhuysen and Pierrot 2020). Only in Brazil is the average income from coffee sufficient to provide farmers with a living income (Cordes et al. 2021; Panhuysen and de Vries 2023). Rural coffee producing regions tend to lack access to affordable and clean energy, good transportation infrastructure, and technology, such as machinery and equipment for coffee production, harvesting, and post-harvest processing, as well as digital tools to build closer relationships with end consumers (Sachs et al. 2019; Santos et al. 2022). Furthermore, coffee farmers often face food insecurity and lack access to clean water, sanitation, social protection, capital, and insurance (Sachs et al. 2019; Panhuysen and Pierrot 2020; Brenes-Peralta et al. 2021). While coffee farmers, especially smallholders, often live in precarious conditions, farm workers and sharecroppers represent the most vulnerable group in the coffee value chain (Panhuysen and Pierrot 2020; Morais and César Pinheiro da Silva 2021; Sigrist et al. in review). Lack of labor rights implementation, exposure to arduous work and health risks, harassment, both sexual and non-sexual, and forced labor are still present in many coffee producing regions (Sachs et al. 2019; Panhuysen and Pierrot 2020; Maguire-Rajpaul et al. 2020; Panhuysen and de Vries 2023). Furthermore, farm workers often lack the right or possibility to collectively organize, and child labor and gender discrimination remain widespread problems (Sachs et al. 2019; Panhuysen and Pierrot 2020).

There are different methods to systematically assess the social impacts of global value chains, such as the coffee value chain. Social Life Cycle Assessment (S-LCA) has recently gained interest and importance to better understand and measure the positive and negative social impacts of products or services on various stakeholders, such as workers and value chain actors, throughout their life cycle (Lucchetti et al. 2018; UNEP 2020; Pollok et al. 2021; Cellura et al. 2024). The most common and recognized S-LCA framework is provided by the United Nations Environment Programme’s (UNEP) Guidelines for Social Life Cycle Assessment of Products and Organizations (UNEP 2020). However, S-LCA is still at a pioneering stage and to our knowledge, there are only three S-LCA studies focusing on coffee. Two including case studies in Costa Rica (Brenes-Peralta et al. 2021) and Indonesia (Rahmah et al. 2023), and one focusing on the Italian coffee sector (Rehman et al. 2025). All three studies are based on the UNEP guidelines (2020). Nevertheless, they differ in their implementation of the guidelines, such as in the selection of stakeholder categories, impact subcategories, and indicators, as well as in the definition of system boundaries. The studies of Brenes-Peralta et al. (2021) and Rahmah et al. (2023) are based on primary data while the study of Rehman et al. (2025) is based on secondary data from the Social Hotspot Database (SHDB) and uses primary data only to contextualize the results. The three studies found different social impacts of coffee production. Brenes-Peralta et al. (2021) identified hotspots in social security and women’s empowerment, and found no evidence of child labor, forced labor, or community health risks. Rahmah et al. (2023) found that a farmer-managed coffee industry led to the most positive social outcomes for most stakeholders. Rehman et al. (2025) identified significant labor condition gaps in origin countries and stressed the need for stronger monitoring and social protection systems.

Unlike the environmental LCA methodology, S-LCA has not yet been standardized. Although the ISO 14,075 standard based on the UNEP guidelines (2020) was published in 2024 (ISO 2024), methodological gaps remain (Tokede and Traverso 2020; Traverso et al. 2025). Main challenges include defining the goal and scope (Lucchetti et al. 2018; Tokede and Traverso 2020), collecting inventory data (Lucchetti et al. 2018; Brenes-Peralta et al. 2021; Chabrawi et al. 2024), measuring social indicators (Tokede and Traverso 2020; Chabrawi et al. 2024), and operationalizing different assessment methods (Brenes-Peralta et al. 2021; Cellura et al. 2024; Chabrawi et al. 2024). Furthermore, there is a lack of studies based on primary data (Chabrawi et al. 2024), insufficient implementation of participatory approaches (Tokede and Traverso 2020; Sehlin MacNeil et al. 2021), and a lack of transparency regarding methodological adaptations and data collection (Tokede and Traverso 2020; Pollok et al. 2021). This also leads to a lack of comparability and reproducibility of results (Pollok et al. 2021; Chabrawi et al. 2024). Additionally, the global coverage of S-LCA studies remains limited, particularly in Africa (Tokede and Traverso 2020; Traverso and Mankaa 2025). Many studies rely on secondary data and generic databases such as the SHDB and the PSILCA database, which provide social risk data at country and sector levels (Chabrawi et al. 2024; Roche et al. 2025). While these databases are commonly used to define social hotspots and prioritize activities, stakeholders, and impacts, they lack detailed, primary, site-specific data and are unable to capture positive impacts (Benoit-Norris et al. 2012; Zimdars et al. 2018; Pollok et al. 2021; Roche et al. 2025). Consequently, the local legitimacy and relevance of such S-LCAs is limited (Sehlin MacNeil et al. 2021), and it is difficult to compare case studies within the same country and sector (Cellura et al. 2024; Rehman et al. 2025). This would, however, be important in the agricultural sector, where production methods (e.g. conventional or organic) and trading relationships (e.g. futures market or direct trade) can vary substantially within countries, as well as within and between agricultural products (Rehman et al. 2025). This is particularly true for products such as coffee, which have a rather high level of market differentiation.

There is a need for studies that clearly and comprehensively document the methodological adaptations and choices (Tokede and Traverso 2020; Pollok et al. 2021; Brenes-Peralta et al. 2021), incorporate primary data and local contextualization (Tokede and Traverso 2020; Chabrawi et al. 2024; Traverso and Mankaa 2025), provide a better description of the sample (Lucchetti et al. 2018; Brenes-Peralta et al. 2021; Chabrawi et al. 2024), and cover a wider range of stakeholder categories and impact subcategories (Zimdars et al. 2018; Tokede and Traverso 2020). Larger sample sizes are also required to produce more robust S-LCAs (Brenes-Peralta et al. 2021; Traverso et al. 2025). Therefore, in this study, we operationalized and applied the S-LCA methodology to assess both positive and negative social impacts of nine coffee case studies in the South of Minas Gerais, Brazil, and in South Kivu, Democratic Republic of the Congo (DRC). We followed a rigorous data collection procedure based on primary, site-specific data and systematically involved different actors at the upstream stages of the coffee value chain, including workers, *metayers*^1^, farmers, and owners and managers of plantations and cooperatives, to capture multiple perspectives (Chabrawi et al. 2024).

## Methods

We operationalized the S-LCA methodology based on the UNEP guidelines (2020) and the ISO standard 14,075 (ISO 2024). We selected the reference scale approach to focus on immediate activities (Brenes-Peralta et al. 2021) and allow for an objective assessment of impacts (Ramirez et al. 2014).

We applied the S-LCA methodology in five case studies in Brazil and four case studies in the DRC, both *Coffea arabica* origin countries. While Brazil maintains its position as the world’s largest coffee producer (FAO 2025), the DRC is regarded as having the greatest agricultural potential in Africa (UNDP 2025). There is a common narrative that the country aspires to become like Brazil in terms of agricultural development (personal communication from actors in the Congolese agricultural sector). However, war, armed conflicts, and violence-driven displacement severely affect not only the population in Eastern DRC but also agricultural and coffee production (Richey and Ponte 2021; Musoko 2022).

**Fig. 1:**
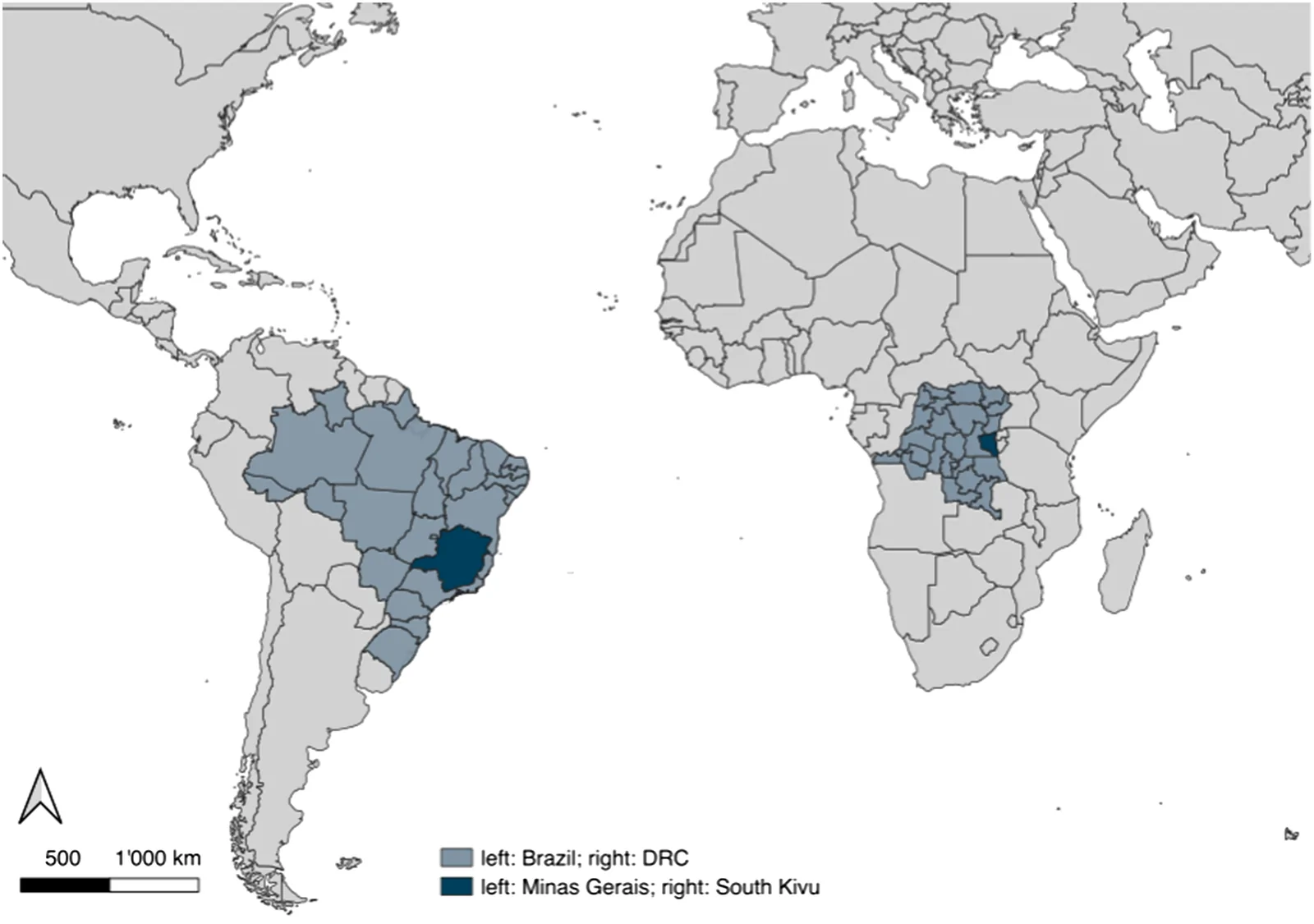
Map of the case study regions showing Minas Gerais, Brazil and South Kivu, DRC

Following exploratory fieldwork in both countries in 2022, we carried out value chain and stakeholder mapping for the case study regions of Minas Gerais, Brazil and South Kivu, DRC (Fig. 1). 

The results of this mapping informed our selection of case studies. The case studies were selected to represent the diversity of production systems and organizational structures across both regions, thereby covering differently organized coffee value chains. Table 1 provides a description of the selected case studies, alongside their respective sample sizes. Thom et al. (2025) and Thom et al. (2026) provide additional qualitative information on the case studies and an evaluation of their environmental impacts.

**Fig. 2:**
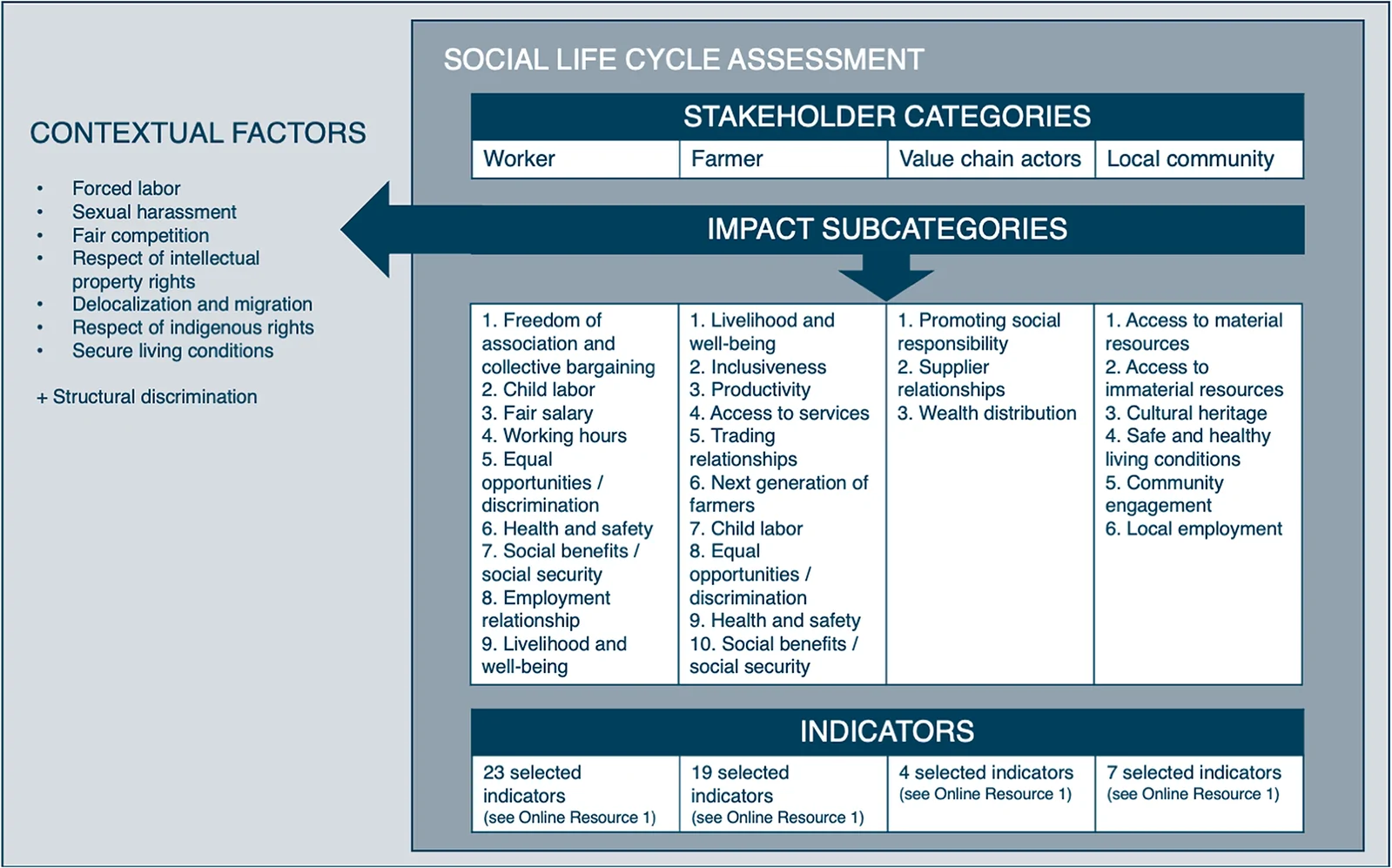
Proposed S-LCA framework with selected stakeholder categories, impact subcategories, and contextual factors

### Goal and scope definition

In this study, the objective was to assess the social impacts of five coffee case studies in Brazil and four in the DRC, comparing case studies within each country and, more broadly, contrasting the two country contexts, by using primary data and adopting the reference scale approach. Brenes-Peralta et al. (2021) identify the farm level as a social hotspot that should receive special attention. Therefore, we defined the system boundaries as cradle-to-gate, covering all actors involved in coffee cultivation and post-harvest processing up to the point where the coffee is ready to sell (i.e. upstream actors). Depending on the case study, this point was either the cooperative or the plantation gate. Our functional unit was the production of *Coffea arabica* beans. As social impacts were assessed using a qualitative reference scale rather than quantified per unit of output, a reference flow (i.e., a specific quantity of coffee production) was not required to conduct this assessment.

#### Selection of stakeholder categories

From the six proposed stakeholder categories by UNEP (2020), we adapted and selected three, namely the categories ‘worker’, ‘value chain actors’, and ‘local community’. In the UNEP guidelines, ‘smallholders including farmers’ are treated as subcategory of the stakeholder category ‘worker’. Given the importance of farmers in this study, we created a separate stakeholder category for them and expanded its scope from smallholders to farmers in general (Brenes-Peralta et al. 2021). The stakeholder category ‘consumer’ was not included because, in line with our system boundaries, our focus was on actors in the upstream stages of the coffee value chain (i.e. the supply chain, covering production and primary processing). The stakeholder categories ‘society’ and ‘children’ were not included because it was not feasible to obtain primary data for these stakeholder categories within the scope of our fieldwork. However, child labor was assessed as part of the stakeholder categories ‘worker’ and ‘farmer’.

#### Selection of subcategories and indicators

We included all impact subcategories for each of the selected stakeholder categories as suggested in the methodological sheets for subcategories in S-LCA (UNEP 2021). Where appropriate we aligned the subcategories of the stakeholder categories ‘worker’ and ‘farmer’ to better compare the two categories (Harmens et al. 2022). Subcategories that cover very sensitive topics or topics that require more specific, higher-level attention were treated as contextual factors. We considered the subcategories ‘forced labor’ and ‘sexual harassment’ to be highly sensitive because they concern potential experiences of exploitation and trauma that interviewees, particularly workers and farmers, may be unable to disclose directly, especially without specialized safeguarding protocols. The other subcategories that we treated as contextual factors, such as ‘respect of intellectual property rights’ and ‘delocalization and migration’, concern issues that require more specific, higher-level attention that we could not adequately capture with the primary data collected through our case studies. We propose a framework that includes these subcategories through secondary data from context-specific scientific and grey literature (see Online Resource 1, S2), rather than primary data, and hence on an aggregated rather than individual level (Ramirez et al. 2014). We chose scientific and grey literature over S-LCA databases because of financial constraints and because database-based assessments risk introducing new biases (Díez-Hernández et al. 2026). We added ‘structural discrimination’ to the contextual factors to account for potentially hidden, country-specific patterns of structural discrimination, including discrimination based on gender, race, and ethnicity (Crenshaw 1994; Raposeiras and Couto 2021; Grant 2021; Franck and Prapha 2021; Sigrist et al. 2025). Figure 2 provides an overview of the proposed S-LCA framework with the selected subcategories for each stakeholder category and the contextual factors.

For all impact subcategories based on primary data, we defined corresponding indicators based on the methodological sheets for subcategories in S-LCA (UNEP 2021) and adapted them to the context of coffee production. Online Resource 2 provides an overview of all indicators, including the original UNEP indicators and explanations of the adaptations made. Depending on the complexity of the impact subcategory, one or several indicators were used to assess it. We selected UNEP indicators based on their relevance to our study context and the feasibility of collecting corresponding primary data. This judgement was informed by insights from the exploratory fieldwork and by exchanges among the co-authors of this study, including researchers from Brazil and the DRC. Additionally, we added new indicators where necessary to better reflect the local context or to address additional relevant topics. Given time and financial constraints, no stakeholder consultation was conducted for the selection of indicators. The final set of indicators comprises 53 indicators, of which 31 are original UNEP indicators and 22 are newly developed or adapted specifically for the coffee sector.

### Social life cycle inventory analysis

The social life cycle inventory (S-LCI) was based on primary data collected through structured face-to-face interviews with 80 workers, 10 *metayers*, 60 farmers, and 20 owners and managers of plantations and cooperatives (see Table 1). The interviews were conducted from April to May 2023 in the DRC, and from October to December 2023 in Brazil. We selected the participants using a purposeful sampling technique based on representing the diversity of functions and roles in the cooperatives and plantations, the interviewees willingness to participate in the study, and their availability (Ramirez et al. 2014; Palinkas et al. 2015). The selection of member farmers of cooperatives was done in collaboration with the staff of the cooperatives. All the data collected was anonymous and participation was voluntary. We did not pay the interviewees but gave them a small gift after the interview to acknowledge the time they contributed, without informing them of this gift in advance to avoid influencing participation or responses. The interviews took place in the interviewees preferred location, usually at home, in a neutral location or at the workplace, however, with sufficient distance from supervisors to ensure comfort and confidentiality. The structured interviews included questions for all indicators of the previously defined impact subcategories, and we collected qualitative, semi-quantitative, and quantitative data. We posed similar interview questions to workers, *metayers*, farmers, as well as to owners and managers of plantations and cooperatives to capture potential variations in responses between these interviewee groups. The interview guidelines used with farmers in Brazil are provided as an example in Online Resource 3. Sociodemographic data of the interviewees and information on their main functions are provided in Table SI 1 and Table SI 2.

### Development of reference scales

To evaluate the inventory data, we developed clear reference scales for each of the 53 indicators, following and adapting the approach of UNEP (2020) and Ramirez et al. (2014), and drawing on examples of Ostos et al. (2024) and Vinci et al. (2025). We defined a reference scale with four levels, each of which corresponds to a performance reference point representing a threshold or objective for defining the level of social performance. The levels range from − 1 to 2, with level 0 representing ‘compliance with local laws/basic societal expectations/common practices in the sector’, level − 1 representing ‘non-compliance’, level 1 ‘proactive efforts/progress beyond compliance’, and level 2 evidence of ‘outstanding efforts/positive outputs achieved/ideal performance’. Unlike the five-level scale suggested by UNEP (2020), we did not further distinguish between different levels of non-compliance, as we considered it neither feasible nor ethically justifiable to rank the severity of non-compliant situations (e.g. different degrees of failing to pay a minimum wage). We did, however, distinguish two levels of performance beyond compliance, to allow for a more differentiated view of performance and to better capture variation across our case studies. Table 2 shows the complete reference scale with an example for the indicator ‘lowest paid worker’, and Online Resource 2 provides an overview of all indicators with their reference scales and respective sources.

Most indicators have reference scales with all four levels, however, where this was not feasible, we developed reference scales with three (-1, 0, 1) or only two levels (-1, 0 or 0, 1). The seven indicators with three-level reference scales represent cases where it was not possible to differentiate between different levels of ‘beyond compliance’ (e.g. having a yield exceeding the country average). The 10 indicators with two-level reference scales represent yes or no questions (e.g. having a certification or not). We acknowledge that the reduced number of levels for these indicators lowers their resolution, meaning that cases falling within the same level cannot be further distinguished from one another. We consider this an acceptable trade-off relative to introducing additional thresholds that could not be defensibly grounded in available evidence.

For the definition of each level, we used the above-mentioned literature and country-specific sources, such as legislation and laws, particularly labor law. We also consulted experts and public officials. Thresholds and the overall framework were further discussed among the co-authors of this study, including researchers from Brazil and the DRC. For indicators linked to the voluntary implementation of additional projects or the provision of additional services, we defined the levels of ‘beyond compliance’ numerically. Level 1 represents the implementation or provision of one or two projects or services, while level 2 represents the implementation of more than two projects or services (Ramirez et al. 2014).

### Social life cycle performance assessment

Using ISO 14,075 terminology, we conducted a social life cycle performance assessment (S-LCPA). We assessed the social impacts by evaluating all indicators across the impact subcategories of each stakeholder category. Primary data collected from interviews was categorized using the country specific four-level reference scales we developed. We assigned a score of -1, 0, 1 or 2 to each interviewee for each of the 53 indicators, by systematically comparing their responses against the reference scale levels, which ensured consistent and accurate assessment. The dataset with the S-LCPA results is provided in an online repository (see data availability).

#### Scoring method

For each interviewee, we created the arithmetic mean of all corresponding indicators per impact subcategory, including scores of -1. Individual interviewee scores were then aggregated to generate a single social performance score per subcategory for each case study and interviewee group (*workers*, *metayers*, *farmers*, *management*). We used R Studio (R Core Team 2024) to first calculate mean scores across indicators at the individual level, and then calculated the arithmetic mean across case studies and interviewee groups. Because averaging can mask individual non-compliant cases, we additionally tracked indicators scored at level − 1 separately, to maintain their visibility regardless of the aggregated mean score. For each impact subcategory, we counted the number of -1 responses per interviewee, then summed these counts across interviewees within each case study and interviewee group. We visualized both the mean scores and the negative counts per case study and interviewee group using heatmaps.

All interviewees, indicators, and impact subcategories were weighted equally in the aggregation process, line with common practice in S-LCA (Ramirez et al. 2014; UNEP 2020; Luna Ostos et al. 2024), as differential weighting would require normative assumptions about the relative importance of indicators for which no methodological consensus currently exists. To support transparency and allow for alternative interpretations, we provide fully disaggregated results at the interviewee and indicator level (see data availability and Online Resource 1, S3).

### Data analysis and interpretation

To analyze the results, we first conducted non-parametric Kruskal-Wallis tests, followed by post hoc Dunn’s tests with Bonferroni correction (*p* < 0.05), to test for differences between case studies. The interviewee group *management* could not be tested because of the small sample size (*n* = 1) in the Brazilian case studies. Therefore, we followed a descriptive analysis and calculated the raw difference in mean performance scores between them. An absolute difference of ≥ 1.0 on the − 1 to 2 performance scale was characterized as a large and substantial difference. We followed the same approach to examine differences between the responses of the *management* and those of other interviewee groups (*workers*, *metayers*, and *farmers*) within the same case study. Given the substantial contextual differences between Brazil and the DRC, statistical comparisons between the two countries are reported to contrast the two contexts rather than to rank performance and should be interpreted with caution.

In February 2025, we conducted six face-to-face semi-structured interviews with case study participants in Brazil to validate our methodology and interpret the results. We conducted one interview with the president of B1, two with farmers of B2, one with the sustainability manager of B2, one with the plantation owner of B4, and one with a worker of B5.

## Results

Our following analysis primarily focuses on comparisons within each country, given the highly context-dependent nature of social performance in S-LCA. Cross-country comparisons are nevertheless reported, however, not to establish a performance ranking but to juxtapose the two contrasting country contexts.

**Fig. 3:**
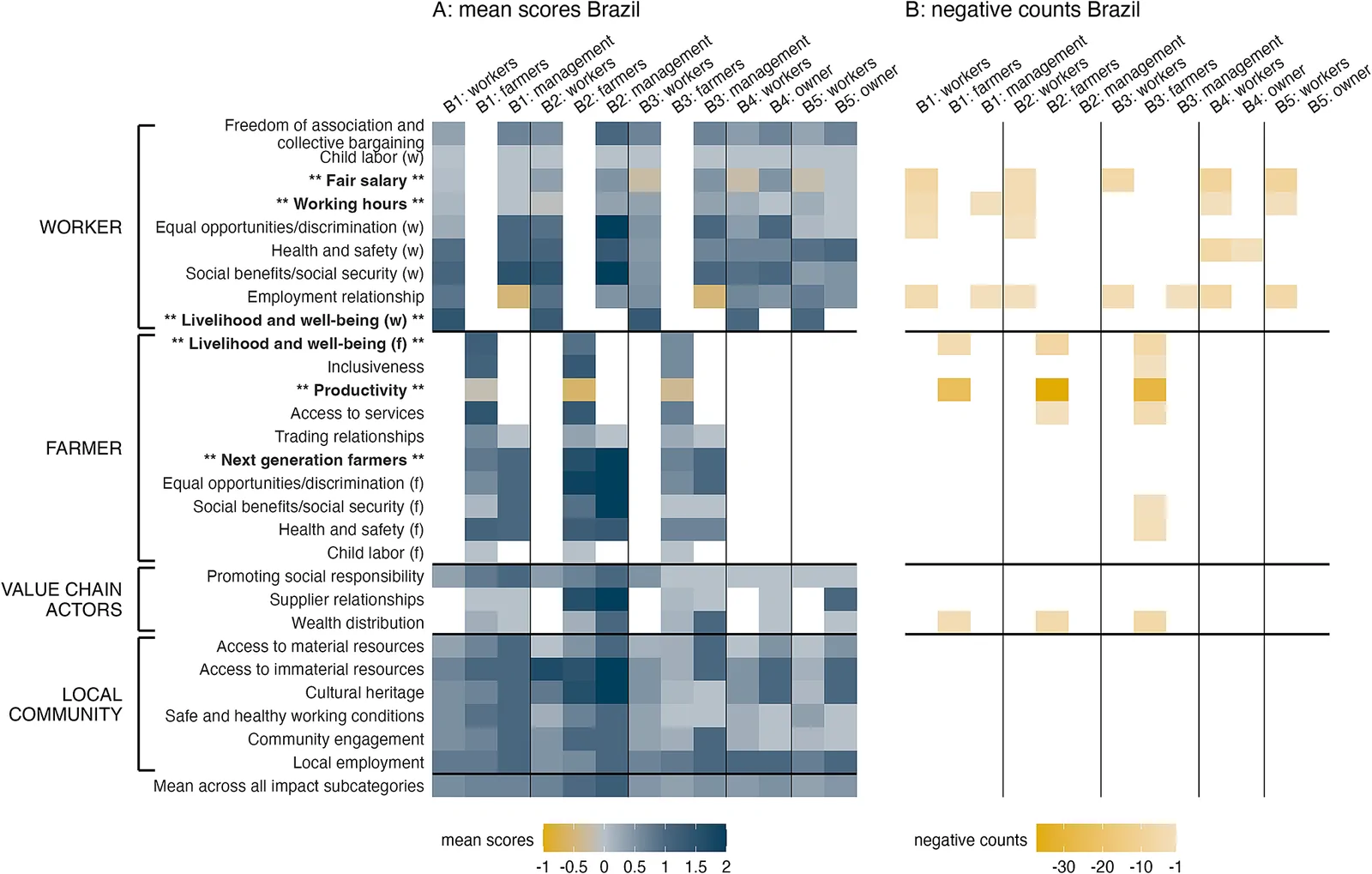
The heatmaps show the social performance of the different case studies in Brazil, divided by interviewee group (workers, farmers, management). The results are displayed for each impact subcategory grouped by the corresponding stakeholder categories ‘worker’, ‘farmer’, ’value chain actors’, and ‘local community’. The x-axis shows the case study and interviewee group, and the y-axis shows the impact subcategories and stakeholder categories. Heatmap A shows the mean performance scores (on the − 1 to 2 reference scale) for each interviewee group, while the heatmap B shows the total number of individual responses scored at level − 1 (negative counts, i.e. non-compliant cases) for each interviewee group. The negative counts are displayed as negative values for visual distinction; the values represent response counts, not performance scores. Subcategories in bold (and embraced by **) are the ones that were ranked highest during the validation interviews

**Fig. 4:**
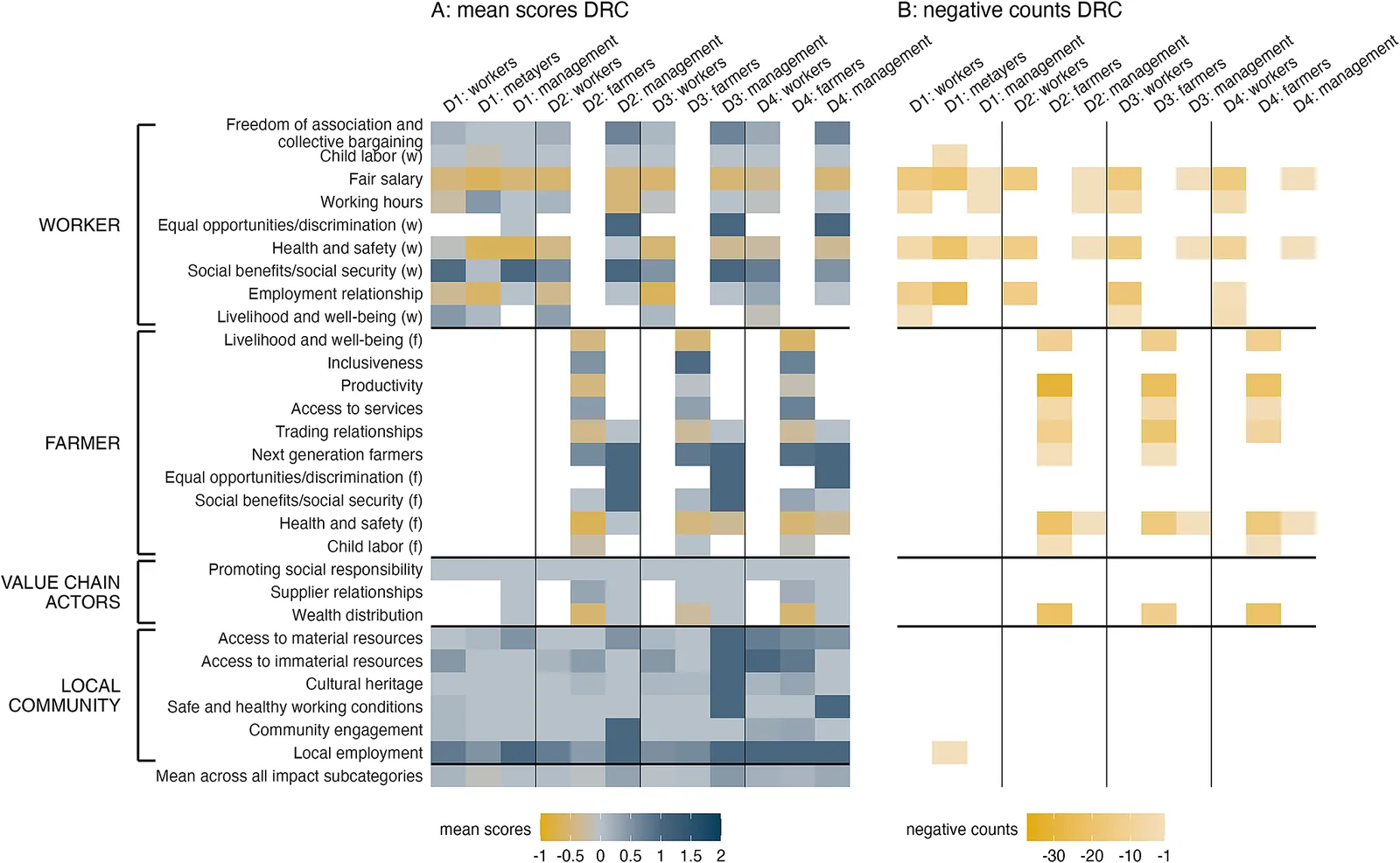
The heatmaps show the social performance of the different case studies in the DRC, divided by interviewee group (workers, metayers, farmers, management). The results are displayed for each impact subcategory grouped by the corresponding stakeholder categories ‘worker’, ‘farmer’, ’value chain actors’, and ‘local community’. The x-axis shows the case study and interviewee group, and the y-axis shows the impact subcategories and stakeholder categories. Heatmap A shows the mean performance scores (on the − 1 to 2 reference scale) for each interviewee group, while the heatmap B shows the total number of individual responses scored at level − 1 (negative counts, i.e. non-compliant cases) for each interviewee group. The negative counts are displayed as negative values for visual distinction; the values represent response counts, not performance scores

Figure 3 shows the social performance of the different case studies in Brazil divided by interviewee group (*workers*, *farmers*, *management*). The mean scores across all impact subcategories and case studies ranged from 0.3 to 0.7 for the interviewee group *workers*, from 0.4 to 1 for the interviewee group *farmers*, and from 0.5 to 1.2 for the interviewee group *management*. Figure 4 shows the social performance of the different case studies in the DRC divided by interviewee group (*workers*,* metayers*,* farmers*, *management*). The mean scores across all impact subcategories and case studies ranged from − 0.09 to 0.2 for the combined interviewee group *workers* and *metayers*, from − 0.04 to 0.1 for the interviewee group *farmers*, and from 0.03 to 0.4 for the interviewee group *management*. Kruskal-Wallis tests and post-hoc Dunn’s tests with Bonferroni correction showed significant differences between case studies from the two countries and between case studies within the same country. We tested for significant differences between case studies both for each impact subcategory individually and for the mean score across all impact subcategories. The results of the tests are provided in Table SI 3 and Table SI 4. Furthermore, substantial differences were found among the *management* across different case studies and between the *management* and other interviewee groups (*workers*, *metayers*, and *farmers*) within the same case study (see Table SI 5 and Table SI 6). In most instances, the Brazilian case studies scored higher (i.e. higher scores on the − 1 to 2 reference scale) than the Congolese case studies, a difference we report to contrast the two contexts rather than to rank performance

The responses of the *management* were generally more positive than those of the other interviewee groups (*workers*, *metayers*, and *farmers*), indicating that the *management* rated the situation more favorably. They were substantially more positive in 4 instances in Brazil and in 9 instances in the DRC. They were only substantially less positive in 2 instances in Brazil and 1 instance in the DRC. The within-country differences are elucidated in the next two chapters. A description of the contextual factors in both countries is provided in Online Resource 1 (S2). Disaggregated results for the different stakeholder categories are provided in Figures SI1, SI2, SI3, and SI4.

### Case studies Brazil

#### Stakeholder category ‘worker’

In the stakeholder category ‘worker’, the mean scores of the Brazilian case studies ranged from 0.4 (B5) to 0.7 (B2) for the interviewee group *workers* and from 0.3 (B5) to 1 (B2) for the interviewee group *management*. High performance was observed in the impact subcategories ‘livelihood and well-being (w)’, ‘social benefits/social security (w)’, ‘health and safety (w)’, ‘equal opportunities/discrimination (w)’, and ‘freedom of association and collective bargaining’, and low performance with most non-compliant cases for ‘fair salary’, ‘working hours’, and ‘employment relationship’. Significant and substantial differences were found in the impact categories ‘social benefits/social security (w)’, ‘equal opportunities/discrimination (w)’, and ‘employment relationship’. Mostly, case study B2 scored highest, and the cooperatives (B1, B2, B3) higher than the commercial plantations (B4, B5) (see Table SI 3 and Table SI 5).

Case study B2 offers a wide range of social benefits and professional development opportunities for its employees beyond what is required by law, explaining its higher scores in this impact subcategory. Employees have access to a health plan covering 70% of the medical costs, gymnastics sessions, and subsidized English classes. Furthermore, they get a day off on their birthday, the monthly food allowances are double the amount required by law (R$^2^ 120–180), and they have additional discounts at supermarkets, hospitals, gas stations, physiotherapy clinics, dental clinics, and pharmacies. The cooperative also provides training in coffee classification and tasting, roasting, and preparing and serving coffee (i.e. barista skills), as well as in topics such as administration, human resources, deliberation, communication, emotional intelligence, recycling, sustainable agricultural practices, and environmental topics. The other case studies offer considerably fewer additional benefits beyond compliance. Case study B1 offers a 14th salary to its employees and money for transport. Furthermore, training in coffee classification and tasting, coffee quality, barista skills, beekeeping, good agricultural practices, and environmental topics. Case study B3 grants holidays for their two employees under RPA^3^ contracts and the social movement offers courses on agricultural, ecological, and social topics. The plantation B4 provides a monthly food basket and coffee to their employees and courses related to the specific fields of work of the employees (e.g. coffee classification, good agricultural practices, or technical training). The plantation B5 offers no additional social benefits to their employees; however, it does offer courses related to the specific fields of work of the employees.

Also, in terms of equal opportunities, case study B2 scored highest. The cooperative has various committees consisting of both employees and cooperative members. They have a gender and equality committee, a complaints committee, and an ethics committee, which address gender-related misconduct and raise awareness of violence against women and femicides. Furthermore, the cooperative also hires pregnant women (which is rarely done in Brazil), and there were no reports of gender-based task divisions. Case study B3 follows the principle of the social movement aiming to always have both a man and a woman in all leadership roles. In case study B1, there were some reports of gendered work tasks, such as female employees having to additionally take over care duties like cleaning the cooperative’s kitchen. However, the cooperative has a project that exclusively sources coffee from female coffee producers. Some employees of case study B4 mentioned that the plantation owner actively promotes women, while in case study B5, there were clear gender-based task divisions.

Most interviewed workers had a working contract, either a Carteira Assinada^4^ or a RPA contract. Only the cleaning lady of case study B1 and a rural/farm worker employed by a farmer of case study B2 worked without a legal contract. Across all case studies, most interviewed workers reported presence of essential contract elements. In case study B1, most contract elements were provided, though some workers reported not receiving additional payments for hazardous tasks or parental leave. Case study B2 provided all elements, with enhanced maternity leave (4 months vs. 3-month minimum required by law) and paternity leave (10 days vs. 5-day minimum required by law) and case study B3 provided a 13th salary to all except the two RPA workers (who received year-end bonuses), with conflicting responses on additional payments for night work and hazardous tasks. Case studies B4 and B5 showed differences between the responses of the management and those of the workers regarding additional payments. Worker satisfaction was high across all case studies. Case study B1 scored highest, followed by B2, while case study B4 scored lowest. In general, workers mentioned positive relationships, development opportunities, and autonomy. In case study B3, one worker reported excessive workload, and some workers of case study B5 suggested food baskets would be helpful.

#### Stakeholder category ‘farmer’

In the stakeholder category ‘farmer’, the mean scores of the Brazilian case studies ranged from 0.4 (B3) to 0.9 (B2) for the interviewee group *farmers* and from 0.5 (B3) to 1.5 (B2) for the interviewee group *management*. The case studies showed overall high, beyond-compliant scores in almost all impact subcategories. The lowest performance with most non-compliant cases was observed in the impact subcategory ‘productivity’. Significant and substantial differences were found in the impact categories ‘next generation of farmers’, ‘equal opportunities/discrimination (f)’, ‘social benefits/social security (f)’, and ‘inclusiveness’. Mostly, case study B2 scored highest and case study B3 lowest (see Table SI 4 and Table SI 5).

Case study B2 has a youth group that actively develops projects for younger generations to promote farm succession. They have, for example, a program called ‘my first field’ in which young people learn how to finance the establishment of a coffee field. The cooperative also provides access to credit for the participants of the youth group. Furthermore, most interviewed farmers want their children to continue the family farm. They see coffee farming as more than simply a job, it represents a way of life they wish to pass on. However, careers outside agriculture are also accepted. Case study B1 has no such youth group, however, they try to occasionally advertise courses and internships to involve the youth, and the cooperative supports young persons who wish to take initiative and start own projects. In case study B3 there are no youth-focused initiatives. However, farmers of both cooperatives want their children to continue working with coffee, although they recognize that this depends on factors, such as market conditions, climate change, and their children’s will. Some reported challenges in engaging youth, as many leave for the cities. While farmers acknowledged that rural life is harder and riskier than urban life, some believe it offers a better quality of life. In case study B3, some see coffee farming as the only viable option, while others prioritize their children obtaining education first.

Gender equality efforts varied across the Brazilian case studies. In case study B2, the women’s group actively promotes female participation through courses, leadership opportunities, and events, with efforts to engage both female members and wives of male members. In case study B1, initiatives include a women-led roasting project, women-specific coffee competitions, and medical tests, though farmers acknowledged ongoing challenges in achieving full equality. Lastly, in case study B3, women hold a majority of leadership roles, with women-specific courses in the movement and a women’s group focused on medicinal plants and natural remedies.

Additional social benefits for farmers beyond what is required by law are limited across the case studies. In case study B2, most farmers have the same health plan as the employees, where the cooperative covers 70% of the medical costs. In case studies B1 and B3, farmers reported no additional benefits and only two mentioned having a health insurance. State benefits mentioned across all cases include retirement pensions and Bolsa Família cash transfers. In case study B3, not all farmers are eligible for retirement pensions due to lack of official land entitlements, resulting in one negative count.

Farmer union membership varied across case studies. In case study B1, half the farmers are union members with one farmer receiving courses. In case study B2, all farmers are union members and in case study B3, all but one are members, with some accessing equipment rentals and paperwork assistance through the farmers’ union.

Regarding land rights, farmers in B1 and B2 either fully own their land or combine owned with rented/shared land. In B3, all but one farmer have official land use rights through either ownership or Use Concession Contracts (CCUs). Both land titles and CCUs serve as official documentation granting recognized land use rights in Brazil.

#### Stakeholder category ‘value chain actors’

In the stakeholder category ‘value chain actors’, the mean scores of the Brazilian case studies ranged from 0 (B4 and B5) to 0.5 (B3) for the interviewee group *workers*, from 0.08 (B3) to 0.8 (B2) for the interviewee group *farmers*, and from 0 (B4) to 1.3 (B2) for the interviewee group *management*. Similar and rather high scores were observed in the impact subcategories ‘promoting social responsibility’ and ‘supplier relationships’. Slightly lower scores, with the only non-compliant cases of this stakeholder category, were observed for ‘wealth distribution’. Overall, the differences between the different impact subcategories were rather small compared to the stakeholder categories ‘worker’ and ‘farmer’. Significant and substantial differences were found in all three impact categories: ‘supplier relationships’, ‘promoting social responsibility’, and ‘wealth distribution’. Mostly, case study B2 scored highest and case studies B3, B4, and B5 lowest (see Table SI 3, Table SI 4, and Table SI 5).

The case studies provide various forms of support for seasonal workers to support social responsibility. Both, case studies B1 and B2 provide facilities for legally required medical tests, with case study B2 additionally covering paperwork costs, inspecting worker accommodations for safety, and offering legal courses on proper employment practices. The management of case study B1 also mentioned to assist seasonal workers’ families with schooling. However, farmers noted challenges including rural/farm workers refusing formal registration and concerns about child labor accusations when women bring children to work. In case study B3, support for workers coming from outside includes assistance with food and housing and the provision of small land plots and information. The president of the cooperative, however, highlighted that the social movement drives most worker support and not the cooperative.

In case study B2, next to general assemblies, cooperative members are organized into smaller groups (nuclei) with monthly meetings between the management of the cooperative and nuclei leaders, who relay information and collect feedback from the members, ensuring continuous dialogue. Case studies B1 and B3 hold general assemblies and meetings, but less frequently. Case studies B4 and B5 are commercial plantations without suppliers, though they act as suppliers when selling through cooperatives or direct trade. The owner of case study B5 participates in cooperative decision-making through meetings, with frequency varying by committee.

Stock and external market prices determine coffee prices in all case studies. In B2 and B3, some members reported that prices are explained to them, and some farmers stated they discuss prices with the cooperative and decide when to sell.

Farmers across all case studies reported perceiving prices as too low at various times between 2021 and 2023. For conventional coffee, farmers perceived prices of R$500-1,000 per bag as bad and R$1,000–1,600 as good. Farmers from case study B2 had slightly higher expectations for good prices of conventional coffee compared to those of case studies B1 and B3. For organic coffee, farmers perceived prices of R$800-1,800 per bag as bad and R$1,700-2,500 as good. Farmers from case study B1 tended toward the lower end of these ranges, defining bad prices at a lower threshold than farmers from case studies B2 and B3. Case study B2 again had the highest expectations for good prices. Between 2021 and 2023, farmers received the highest prices in 2022 and lowest in 2021 across all case studies, which is in line with stock prices. Overall, B2 received the highest mean prices.

#### Stakeholder category ‘local community’

In the stakeholder category ‘local community’, the mean scores of the Brazilian case studies ranged from 0.2 (B5) to 0.6 (B2) for the interviewee group *workers*, from 0.3 (B3) to 1 (B2) for the interviewee group *farmers*, and from 0.6 (B4 and B5) to 1.3 (B2) for the interviewee group *management*. High scores were observed in the impact subcategories ‘access to immaterial resources’, ‘local employment’, and ‘cultural heritage’, and lower scores for ‘safe and healthy working conditions’, and ‘access to material resources’. Differences between the impact subcategories were rather small, and overall performance was higher than for the other stakeholder categories. Significant and substantial differences were found in the impact categories ‘access to immaterial resources’, ‘cultural heritage’, ‘community engagement’, and ‘safe and healthy working conditions’. Again, case study B2 scored highest. Case studies B3, B4, and B5 scored lowest and overall, the cooperatives (B1, B2, B3) scored higher than the commercial plantations (B4, B5) (see Table SI 3, Table SI 4, and Table SI 5).

In one way or another, all case studies support infrastructure, social or cultural projects for the community. Case study B2 has the highest number of established projects. They support ecological septic systems, internet access, erosion-prevention terraces, and access to credits for solar panels for members. Additionally, they have numerous community initiatives including supporting vulnerable individuals, organizing ecological walks and recycling projects, donating food and coffee to institutions and people in need, and running sports projects and a community garden. They organize ethical community-led tourism, maintain a worker on the Municipal Cultural Council, safeguard historic churches, participate in traditional events such as Companhia de Reis, and members produced a music recording. Case study B1 provides coffee roasting services to the community, manages the local post office, installed public trash bins, runs trash collection initiatives with schools, assists kindergartens and elderly homes, operates a community garden supplying vegetables to schools and hospitals, and offers community coffee classification courses. They promote culture through tourism initiatives, organize an annual festival celebrating coffee and regional traditions, and recently produced video documentation of producer histories. Case study B3 established plant nurseries, provides weekly vegetable donations to schools, supports gardening projects, and partners with the social movement to host various festivals. Case study B4 supports the school, implemented a beekeeping project, has projects to offer support to women and Afro-descendant coffee producers, participates in regional festivals, and maintains a hotel recognized as a cultural landmark. Case study B5 holds a project collecting and selling coffee to support women and supports cultural activities through collaboration with the House of Culture.

Some case studies are also actively involved in topics around community health. Case study B2 implements various health initiatives, including pesticide blood screenings available community wide. Although their educational offerings are publicly available, the management reported limited attendance, with one farmer highlighting transportation barriers for some community members. While case study B1 offers *Outubro Rosa*^5^ health screenings only to members’ wives and daughters due to high costs, the more affordable *Novembro Azul*^6^ tests are available to all community members. Furthermore, training programs in first aid and equipment use are publicly accessible. Case study B3 works with the social movement on initiatives addressing agroecology, healthy diet, and natural medicine preparation. In case study B4, workers mentioned SENAR^7^ trainings to be publicly accessible, though the owner indicated training typically serves only staff. However, future plans include allocating spots for workers’ family members and local residents. In case study B5, workers gave conflicting reports about community access to trainings. Some indicated public availability while others reported exclusion. The owner clarified that SENAR-organized trainings welcome outside participants, while trainings organized by the plantation are restricted to employees.

### Case studies DRC

#### Stakeholder category ‘worker’

In the stakeholder category ‘worker’, the mean scores of the Congolese case studies ranged from − 0.2 (*metayers* D1) to 0.06 (D4) for the combined interviewee group *workers* and *metayers* and from − 0.02 (D1) to 0.2 (D3) for the interviewee group *management*. The highest performance was observed in the impact subcategories ‘equal opportunities/discrimination (w)’, ‘social benefits/social security (w)’, and ‘freedom of association and collective bargaining’, and the lowest performance with most non-compliant cases for ‘fair salary’, ‘health and safety (w)’, and ‘employment relationship’. *Metayers* from case study D1 scored significantly higher than workers from case studies D1, D3, and D4 in the impact subcategory ‘working hours’, and significantly lower than workers from case study D1 for ‘social benefits/social security (w)’. Furthermore, the management of case study D1 scored substantially lower than that of case studies D2, D3, and D4 in the impact subcategory ‘equal opportunities/discrimination (w)’ (see Table SI 3 and Table SI 5).

The legal working time in the DRC cannot exceed eight hours per day and 45 h per week. While cooperative and rural/farm workers usually work at least this amount of time (with non-compliant cases for workers in case studies D1, D3, and D4), the *metayers* in case study D1 only have to work for five to six hours per day, explaining their higher scores in this impact subcategory. Regarding days of holidays there were some conflicting responses. While one worker from case study D2 reported receiving holidays, the management indicated that paid holidays are not provided to any employees. When applicable (for permanent employees), there were reports of not receiving the legally required holidays in all case studies.

*Metayers* in case study D1 receive no social benefits or professional development opportunities, while the workers in case study D1 receive a meal and potable water, sometimes support with covering health expenses, and access to trainings in good agricultural practices, coffee quality, environmental topics, and, for some workers, administration and bookkeeping. While the management of case studies D2 and D3 also reported providing meals and potable water, only few workers confirmed this. In case study D4 this was the other way around, some workers reported receiving meals and water, while the management did not report this. However, they all offer trainings in good agricultural practices, coffee processing and quality, and, depending on the case study, in other topics such as organic farming or coffee tasting. Here it is important to mention that the trainings are often offered through collaborations with NGOs.

While case study D1 does nothing to promote equal opportunities, all cooperatives reported to do so. Case study D2 reported following a gender policy in the cooperative and treating everyone equally. When hiring, they first check the skills of the candidates but then give priority to women. Case study D3 reported giving everyone a chance who fits the profile they are searching for and sometimes giving priority to women. Finally, case study D4 tries to promote women to work in the cooperative.

#### Stakeholder category ‘farmer’

In the stakeholder category ‘farmer’, the mean scores of the Congolese case studies ranged from − 0.1 (D2) to 0.1 (D3 and D4) for the interviewee group *farmers* and from 0.3 (D4) to 0.6 (D2) for the interviewee group *management*. The highest performance was observed in the impact subcategories ‘equal opportunities/discrimination (f),’ ‘next generation of farmers’, and ‘inclusiveness’. A performance below compliance was observed in the impact subcategories ‘livelihood and well-being (f)’, ‘health and safety (f)’, ‘productivity’, ‘trading relationships’, and ‘child labor (f)’. There were no significant differences among the case studies. However, the management of case studies D2 and D3 scored substantially higher than that of case study D4 for ‘social benefits/social security (f)’.

The management of case study D4 reported not to provide any social benefits to their farmers. The management of case study D2 on the other hand mentioned to distribute coffee seedlings to their members and occasionally prefinance agricultural activities for some of them. The management of case study D3 mentioned the distribution of bean seeds and a program to distribute food and livestock to producers. However, the cooperatives offer other services to their members, such as access to microcredits or trainings. The cooperative of case study D4 successfully established village savings and credit associations called AVECs^8^ among its members, in which the members save money and provide each other with microcredits. Most interviewed farmers of case study D4 mentioned to be associated to an AVEC and have access to microcredits. The management of case studies D3 and D2 mentioned facilitating access to microcredits for their members, case study D3 through the microcredit company PAIDEK^9^. However, not all farmers of these case studies mentioned to have access to microcredits.

In all three cooperatives farmers have access to trainings in topics related to good agricultural practices, such as the production and application of compost, pruning, or intercropping. The management of case study D3 mentioned a training center where women have access to sewing trainings, and the management of case study D4 mentioned a center for alphabetization, so their members, especially the cashiers of the saving groups, can learn how to read and write.

#### Stakeholder category ‘value chain actors’

In the stakeholder category ‘value chain actors’, the mean scores of the Congolese case studies were 0 for the combined interviewee group *workers* and *metayers* in all case studies. They ranged from − 0.08 (D2) to -0.1 (D4) for the interviewee group *farmers* and were 0 for the interviewee group *management* in all case studies. The highest performance was observed in the impact subcategory ‘supplier relationships’ and the lowest, with the only non-compliant cases, for ‘wealth distribution’. There were no significant or substantial differences among the case studies. However, like in Brazil, stock and external market prices determine coffee prices, and cooperatives base their prices on these without involving their members. Most farmers across all the case studies reported perceiving prices in the years 2020–2022 as bad. Only one farmer from case study D3 reported perceiving the prices in the years 2020–2022 as good. For the year 2022, there were 11 farmers who perceived prices as neither good nor bad, for the year 2021 only three did so, and for the year 2020 only one. Farmers reported that prices of 500–2000 CF^10^ (mean 825) per kg of fresh coffee cherries are bad and 1000–10000 (mean 2930) CF per kg of fresh coffee cherries would be good. Farmers from case study D3 had the highest expectations for good prices, and they also received the highest prices in all three years. In line with stock prices, farmers received the highest prices in 2022 and the lowest in 2020 during this period (2020–2022).

#### Stakeholder category ‘local community’

In the stakeholder category ‘local community’, the mean scores of the Congolese case studies ranged from 0.1 (*metayers* D1) to 0.5 (D4) for the combined interviewee group *workers* and *metayers*, from 0.1 (D3) to 0.5 (D4) for the interviewee group *farmers* and from 0.3 (D1) to 0.8 (D3) for the interviewee group *management*. The highest performance was observed in the impact subcategory ‘local employment’, even though the only non-compliant case of this stakeholder category was also in this impact subcategory. The lowest performance was observed in the impact subcategory ‘community engagement’. Like for the Brazilian case studies, differences between the impact subcategories were rather small, and overall performance was higher than for the other stakeholder categories. Significant and substantial differences were found in the impact categories ‘access to material resources’, ‘access to immaterial resources’, ‘cultural heritage’, and ‘safe and healthy working conditions’. Mostly, case study D4 and D3 scored highest and case study D1 lowest, especially the *metayers* (see Table SI 3, Table SI 4, and Table SI 5).

All case studies support some infrastructure, social, or cultural projects for the community. Case study D4 has the highest number of established projects. They invested in repairing standpipes in four villages, run a vocational training center for dressmaking open to the community (including mentoring orphans and unmarried women), run literacy programs, established village savings and credit associations (AVECs) across all villages where members are located, mentor young people in managing agroforestry and tree nurseries, and raise awareness about setting up community vegetable gardens and reforestation. The cooperative distributes information through churches and invites religious leaders and education authorities to participate in activities. Case study D3 did road rehabilitation in some places and provided access to water sources in two villages. They supervise students from agricultural schools during internships, conduct training programs on good agricultural practices with the entire community, run an ecotourism program, and promote arts such as basket weaving. Furthermore, they have a program that re-integrates former armed combatants into the community through the cooperative. Case study D2 rehabilitated a road (with support from local church youth), established coffee washing stations in villages creating employment opportunities for young people and women, distributes coffee seedlings to ensure crop sustainability for future generations, and organizes women’s cultural days teaching about coffee culture. Case study D1 provides limited community support, including occasional seed and livestock distribution, assistance with children’s education and house construction for some workers, and occasional employment opportunities on the plantation for nearby church members.

Case studies D3 and D4 are also partially involved in topics around community health. They both reported offering occasional community trainings about diseases caused by dirty water and dirty hands and the prevention of endemic diseases such as Covid-19. Like the trainings on good agricultural practices, health-related trainings are often offered through collaborations with NGOs.

### Validation interviews

During the six validation interviews in Brazil, we discussed the developed reference scales, perceived most important social impacts and impact subcategories, and preliminary S-LCA results. In general, the developed reference scales and methodology were approved by the interviewees. Most disagreement emerged regarding the adequacy of the living wage and living income. Some considered it as reasonable, while others still perceived it as too low to have a good quality of life, especially if major healthcare or educational costs occur. The interviewees ranked ‘livelihood and well-being’, ‘fair salary’, ‘working hours’, ‘productivity’, and ‘next generation of farmers’ among the most important social impact subcategories, though they emphasized the interconnected nature of these issues and the difficulty of ranking them (Fig. 3). Additional concerns raised beyond the predefined impact subcategories included timing for coffee sales, certification complexity and costs, labor shortages due to rural-urban migration, climate change impacts, and the critical role of cooperatives in providing technical support and education to farmers.

## Discussion

### Social performance of coffee case studies in Brazil and the DRC

#### Regional differences

The overall higher social performance scores of the Brazilian case studies compared to the Congolese case studies – reported here to contrast the two contexts rather than to rank performance – can be interpreted as reflective of the very different socio-economic and political situations in the two countries. Brazil is the only coffee origin country where farmers predominantly earn a living income, mostly due to larger farm sizes and higher productivity compared to the global average (Cordes et al. 2021; Panhuysen and de Vries 2023). Furthermore, worker wages are higher, and labor legislation is the strictest and most advanced among coffee origin countries (Cordes et al. 2021; Santos et al. 2022; Medeiros Guimarães et al. 2022; Panhuysen and de Vries 2023). However, Brazil also has severe land inequality issues, which among other things drive the social movement’s (B3) struggle for land redistribution (Fernandes 2013; Dolce 2020). Additionally, enforcement of labor legislation remains a challenge, and forced labor and slavery-like working conditions exist (Hjerl Hansen et al. 2016; Cordes et al. 2021; Nakaharada et al. 2022; Perosa et al. 2024; Ministério do Trabalho e Emprego 2025). In the DRC, legacies of colonialism and deliberate ‘underdevelopment’ by European colonial regimes (Rodney 1972), have created extremely precarious living conditions and severe social impacts (Richey and Ponte 2021; Slosse et al. 2023). The contextual factors are precarious and exacerbated by 30 years of war and armed conflict and the withdrawal of USAID funding, with USAID formerly functioning as a key actor in coffee value chain development (Richey and Ponte 2021; Slosse et al. 2023). It is very well possible that the social situation further deteriorated since the re-escalation of war and armed conflict in January 2025.

Our findings show that the context strongly influences the social performance of our coffee case studies. However, we also found clear differences between case studies in the same country, showing that not only regional context but also organization matters.

#### Stakeholder categories

In the Brazilian case studies, mean performance scores were highest for the stakeholder category ‘farmer’, followed by ‘local community’, ‘worker’, and lastly ‘value chain actors’. In the Congolese case studies this was similar, mean performance scores were highest for the stakeholder category ‘local community’, followed by ‘farmer’, ‘worker’, and lastly ‘value chain actors’. The comparatively high scores of the stakeholder categories ‘farmer’ and ‘local community’ can probably be attributed to the nature of cooperatives, especially certified cooperatives (strongly present in our sample), that usually support their members and their surroundings, form alliances with other actors, engage with communities, and provide local employment (Meemken et al. 2019, 2021; Brenes-Peralta et al. 2021; Rahmah et al. 2023). High performance in the ‘local community’ stakeholder category was also found in an S-LCA in Colombia, attributable to the involvement of various actors and their collaborations with the local community (Luna Ostos et al. 2024). The comparatively low performance of ‘value chain actors’ supports existing literature showing that there is great inequality between the different actors in the coffee value chain, with downstream actors in importing countries receiving the largest share of the value, while most farmers live below the poverty line (Grabs and Ponte 2019; Elsby 2020; Cordes et al. 2021; Rehman et al. 2025). Furthermore, our findings are in line with literature about working conditions, showing that rural/farm workers and sharecroppers are often exposed to even more precarious conditions than smallholder farmers (Panhuysen and Pierrot 2020; Morais and César Pinheiro da Silva 2021; Sigrist et al. in review).

In general, there seems to be a mismatch between social performance and priorities of affected actors. The impact subcategories showing the lowest performance, such as ‘fair salary’, ‘livelihood and well-being’, and ‘productivity’, are the ones that were ranked highest during the validation interviews and were also shown by previous research to be most important for farmers and workers (Thom et al. 2025). This mismatch underscores the relevance of these impact subcategories despite our use of equal weighting in the display of the results and suggests that a stakeholder-informed weighting scheme could further emphasize these performance gaps in future assessments.

#### Impact subcategories

In contrast to literature, we found a high performance for the stakeholder category ‘worker’ in the impact subcategories ‘freedom of association and collective bargaining’ and ‘social benefits/social security (w)’. Sachs et al. (2019) highlight the right of rural coffee workers around the world to organize and form unions as challenging. In our study, we found no non-compliant cases, meaning that there was no evidence that workers cannot organize or cannot be part of unions. However, even the presence of unions or involvement in basic decision-making does not always ensure that the worker’s issues are sufficiently addressed (which would require a social performance score of 2). This gap matters in practices: the presence of unions does not guarantee that worker’s everyday concerns, such as wage disputes or unsafe working conditions, are actually resolved through these channels.

Similarly, we found rather high scores for the stakeholder category ‘farmer’ in the impact subcategory ‘inclusiveness’, which also involves the participation in farmer unions or associations that can be beneficial for farmers (Bizikova et al. 2020). Brenes-Peralta et al. (2021) came to similar results, however emphasizing that freedom of association is not enough if there are no efforts to establish formal unions that can act if conflicts emerge. In our case studies, social benefits were present for both, workers and farmers. With a single non-compliant case and several positive scores, we can conclude that all case studies mostly follow the law, and most of them offer small additional benefits, such as access to trainings. This, however, does not ensure social security, which for example does not exist in the DRC. For workers and farmers there, this means that illness, injury, or old age can directly lead to loss of income and precarious conditions. The lack of social protection for coffee farmers and workers has been highlighted by several scholars (Sachs et al. 2019; Brenes-Peralta et al. 2021; Rehman et al. 2025). Brenes-Peralta et al. (2021) found that social security or the lack of it is one of the greatest concerns for coffee farmers and workers in Costa Rica.

The Brazilian case studies also showed high scores in the impact subcategory ‘health and safety (w)’, while the Congolese case studies showed a low, mostly non-compliant scores in the latter. Similar to our Brazilian case studies, Brenes-Peralta et al. (2021) also did not find any health risks in their coffee S-LCA. However, labor health risks are common in coffee production and include injuries from sharp tools and repetitive movements, heavy lifting, pesticide exposure and poisoning, and sun and heat exposure (Sachs et al. 2019; Panhuysen and Pierrot 2020; Sigrist et al. in review). Furthermore, the majority of coffee workers worldwide has no access to healthcare or health trainings (Rehman et al. 2025). This pattern was especially pronounced in our Congolese case studies, where no public healthcare system exists, meaning that workers (and farmers) must rely on private, often unaffordable care or forgo treatment altogether. In Brazil, by contrast, a public healthcare system exists, providing at least a basic safety net even in cases where employer-provided health and safety measures are insufficient. Similarly, we found high scores for ‘health and safety (f)’ in the stakeholder category ‘farmer’ for the Brazilian case studies and a low, mostly non-compliant scores for the Congolese case studies.

In both countries, rather high scores could be observed for ‘equal opportunities/discrimination’ in both stakeholder categories ‘worker’ and ‘farmer’. This is in stark contrast to the outcomes of our literature review of the contextual factors (see Online Resource 1, S2), and to other literature showing that gender discrimination, gendered tasks, unequal pay, and sexual harassment remain widespread problems in the coffee sector globally (Borrella et al. 2015; Sachs et al. 2019; Pereira and Ferreira 2022; Panhuysen and de Vries 2023; Sigrist et al. in review; Sigrist et al. 2025). Two coffee S-LCA studies also found hotspots in gender discrimination and women’s empowerment (Brenes-Peralta et al. 2021; Rehman et al. 2025). This discrepancy between our scores and the wider literature is therefore likely a reflection of what our data collection method could capture, namely the existence of formal gender equity measures, rather than evidence that gendered inequalities in daily farm and household life have been resolved. We mainly collected data about the presence of projects to promote gender equality. Although it is remarkable that most case studies had implemented some projects or measures, likely due to the strong presence of certified cooperatives in our sample, we did not always find evidence that these projects effectively make a difference (which would be expressed with a social performance score of 2). Furthermore, it was beyond the scope of this study to collect primary data on individual experiences of sexual harassment and discrimination, which remain issues in both countries.

Rather high scores could also be observed for the stakeholder category ‘farmer’ in the impact subcategory ‘access to services’, which can mainly be attributed to the provision of trainings. In all case studies, farmers had access to trainings, mainly about topics related to good agricultural practices. In line with findings from literature, access to finance was an issue, with most limited access in the case studies D2, D3, and B3 (Sachs et al. 2019; Panhuysen and Pierrot 2020). Limited access to credit constrains farmer’s ability to invest in their farms or absorb shocks such as poor harvests or price drops.

In all case studies, most farmers want their children to continue coffee farming and most case study cooperatives have some kind of initiatives to promote this, however, the interest of the children is not always given, leading to potential farm succession challenges (Santos et al. 2022).

We found the lowest performance for the stakeholder category ‘worker’ in the impact subcategory ‘fair salary’ and ‘employment relationship’. In line with other studies, we found that workers in our case studies mostly do not earn living wages in the coffee sector (Panhuysen and de Vries 2023), with only seven exceptions in the Brazilian case studies and one in the Congolese case studies. In a S-LCA about the Italian coffee supply chain, Rehman et al. (2025) found that 34% of the workers earn a salary that is below the minimum wage. Unlike in the Congolese case studies, where 71% of the workers earned a salary below the minimum wage, no workers in the Brazilian case studies fell below the legal minimum. However, the legal minimum wage is insufficient to cover basic living expenses in Minas Gerais (Medinaceli et al. 2023), a finding that is consistent with a S-LCA of the chocolate industry in Colombia and other literature (Anker 2006; Hodgetts et al. 2022; Luna Ostos et al. 2024). In practice, this means that even workers who are formally compliant with the law may struggle to meet basic household needs, particularly in the DRC, where the combined absence of living wages and social security leaves workers with little to no buffer against economic shocks.

For the stakeholder category ‘farmer’, a low performance was observed in the impact subcategory ‘productivity’. Most farmers in all case studies had yields below the region’s average and furthermore, especially in the Brazilian case studies, they were unsatisfied with their yields. Except for ‘productivity’, the Brazilian case studies showed satisfactory performance in all other impact subcategories in the stakeholder category ‘farmer’, which, similar to the findings of Brenes-Peralta et al. (2021), is probably attributable to the generally enabling contextual conditions and rather robust national institutions. The mostly non-compliant performance of the Congolese case studies for the stakeholder category ‘farmer’ in the impact subcategories ‘livelihood and well-being (f)’, ‘trading relationships’, and ‘child labor (f)’ is in line with literature showing that incomes from coffee production are not enough to support farmers’ livelihoods, trading relationships are unequal and untransparent, and child labor remains a widespread problem in global coffee production (Sachs et al. 2019; Grabs and Ponte 2019; Panhuysen and Pierrot 2020). Taken together, these findings point to structural vulnerabilities in the Congolese case studies, where low and unstable incomes leave limited room to refuse unfavorable trading relationships or to keep children in school rather than contributing to farm labor.

Overall, case study B2 scored highest in Brazil and case studies D3 and D4 highest in the DRC. While case study B2 is a well-established Fairtrade certified cooperative, the case studies D3 and D4 are both located in a region much less affected by armed conflicts and war than the regions where the other two case studies are located. We also found that the group of *metayers* is the most vulnerable group, suggesting that precarious working arrangements can have additional effects on the vulnerability of workers in the coffee value chain. *Metayers* typically farm land they do not own, which limits their bargaining power and ties their livelihood directly to factors, such as yield fluctuations or landowner decisions, over which they have little control.

Furthermore, especially in Brazil, it seems that the cooperative models score higher than the commercial plantations. Case study B3, which showed lower social performance scores than the other two Brazilian cooperatives, is also the only case study with land tenure issues, instances of prosecution, and cooperative members living in precarious situations. In some cases, land ownership remains contested, with effects in other areas as well, such as the impossibility to promote coffee to the younger generation and challenges in officially certifying and selling coffee.

### Contextual factors

Beyond the impact subcategories assessed with primary data, our review of contextual factors (see Online Resource 1, S2) highlights further social risks in our study regions that our assessment based on primary data could not directly capture. In Brazil, reported cases of forced labor are common in the municipalities where our case studies are located, particularly in the agricultural sector (Hjerl Hansen et al. 2016; Ministério do Trabalho e Emprego 2025) and one worker’s account of a previous work experience illustrates this very well:

The beds were made of grass. We didn’t have running water, a filter to drink water, or a toilet. There was a toilet outside. And everyone slept together. If you were hungry, you stayed hungry because there was no stove to cook on. Or you’d buy a small pot to make coffee and eat cookies. (Rural farmworker, B3)

However, we did not find any indications of forced labor in our case studies and none of the farmers or plantations appeared on the Brazilian Government’s latest ‘dirty list’ of slave labor (Ministério do Trabalho e Emprego 2025).

For the DRC, we found no reports of forced labor in the literature, though this most likely reflects a lack of documentation rather than its absence, as can be assumed based on this coffee sorter’s report on the precarity of her employment:

We don’t eat during the day, not even a sugar cane, you sometimes leave the house without eating anything, you spend the day without eating anything until 3pm when you come home again. (Worker, D3)

Structural racial and intersectional discrimination in the Brazilian coffee sector also remains comparatively undocumented despite its relevance (Crenshaw 1994; Raposeiras and Couto 2021). People who identify as non-white make up more than half of the rural/farm workforce, however, they rarely own large farms or lead cooperatives (Belandi 2019). Furthermore, in our own sample, white workers were more likely than non-white workers to earn above the minimum wage.

Indigenous communities, present in both countries, remain vulnerable: in Brazil, due to agribusiness land expansion (Ferrante et al. 2025), and in the DRC, due to weak legal protection, particularly for Batwa groups (International Alert 2025). Although present in our study regions, they were not involved in any of our case studies, and we could not determine whether this reflects their own choice or systemic exclusion.

Finally, decades of armed conflict in eastern DRC have driven forced internal displacement, and most metayers of case study D1 are internal migrants who arrived with few alternatives than farming on other people’s land.

Together, these findings suggest that our approach based on primary data from specific case studies cannot fully capture all vulnerabilities experienced in the study regions, underscoring the value of complementing the assessment with contextual and qualitative evidence.

### Methodological insights and limitations

This study presents a novel approach for achieving greater case sensitivity in S-LCA through primary data and context-specific reference scales, while adhering to the UNEP guidelines (2020) and ISO standard 14,075 (ISO 2024), thereby contributing to standardized S-LCA studies. Having developed and aligned our reference scales to two markedly different coffee-producing contexts, we consider our context-specific reference scales potentially transferable to other coffee-producing contexts, subject to local testing, adaptation, and validation.

The differences we found between case studies in the same country/region underline the importance of greater case sensitivity in S-LCA that goes beyond generic databases assessing social performance at country and sector levels (Cellura et al. 2024; Roche et al. 2025; Rehman et al. 2025). In this study, we identified differences between case studies located in similar areas and with similar certifications (e.g. B1 and B2). Based on our findings, we suggest that country or sector level assessments risk having a pro-forma nature with several limitations. For example, companies operating in countries or sectors with low social risks could use this to ‘free-ride’. At the same time, companies operating in countries or sectors with high social risks cannot be rewarded for positive efforts, even though these are precisely the contexts where such efforts could potentially have the greatest impact. High-level approaches make it impossible to learn from context-specific insights and to provide guidance on what could be improved, where, and how. Furthermore, existing databases are expensive and hence not widely accessible, hindering research as well as making it difficult to review studies and ensure accountability based on such databases. This highlights the importance of context-specific primary data (Tokede and Traverso 2020; Chabrawi et al. 2024; Traverso and Mankaa 2025; Rehman et al. 2025).

The differences we found between the responses of the management and those of farmers and workers underlie the importance of involving different perspectives and actors of the coffee value chain to obtain reliable results, a finding that is most likely valid for other supply chains as well (Ramirez et al. 2014; Sehlin MacNeil et al. 2021; Chabrawi et al. 2024; Traverso and Mankaa 2025). Systematically interviewing multiple interviewee groups per case study, rather than relying on a single informant type or on company documents and reports as is common in most existing S-LCA studies (Luna Ostos et al. 2024; Traverso and Mankaa 2025; Rehman et al. 2025), constitutes a further methodological contribution of this study. This is particularly relevant given that the responses of the management in our study were in general more positive than those of other interviewee groups, meaning that relying on a single informant type risks systematically overestimating social performance.

We also consider it a methodological contribution to provide disaggregated results and separately display non-compliant cases, in order to avoid their invisibilization through means, which would have occurred in our study.

Our findings reveal several general limitations of the S-LCA methodology. The impact subcategories and indicators suggested by the UNEP guidelines (2020) work best for well-established companies or cooperatives in contexts with clear governmental regulations (Sigcha et al. 2024), revealing a rather corporate and Western-centric approach. The applicability becomes more challenging when working with smallholder farmers and assessing less institutionalized cases, such as case study B3 with its links to a social movement, or when working in countries such as the DRC, where certain elements, like workers’ unions in the agricultural sector, simply do not exist.

The main limitation of this study was the difficulty of reaching seasonal and day laborers employed by farmers, a particularly vulnerable but often excluded group (Meemken et al. 2019; Dedieu et al. 2022). Both data collection sessions took place outside the coffee harvesting season in order to better approach interviewees and avoid additional workload during the harvest. The disadvantage of this approach was that no seasonal farmworkers were present in the Brazilian case studies, and very few farmworker day laborers were present in the Congolese case studies. Especially in Brazil, where these workers often come from different regions, we had no chance of including them in the study, and their exclusion may have led to an overly positive assessment of the social situation. Furthermore, approaching workers always requires a certain dependency on their employer, reflecting existing power asymmetries (Sigrist et al. in review). Employers need to give permission and contacts to systematically approach their employees. Therefore, in both countries, wherever possible, we interviewed workers who were not suggested by the employer and, in some cases, not even mentioned by them.

The treatment of subcategories that cover highly sensitive topics, namely ‘forced labor’ and ‘sexual harassment’, as contextual factors (see Fig. 4) poses another limitation of this study. Our review of contextual factors indicates that both issues are present in the study regions; however, our approach does not allow us to determine whether they also occurred within our specific case studies. In other words, while we are aware of these issues at a broader contextual level, we remain blind to their presence, or extent, within our case studies, due to the lack of primary data on these topics. The only partial exception is ‘forced labor’ in the Brazilian case studies, for which very specific secondary data are available. Collaborations with researchers experienced in working with distressing or traumatic experiences would be beneficial for a comprehensive S-LCA but would require a study of its own.

Finally, the lack of stakeholder consultation for the selection of impact subcategories and indicators led to a final set of indicators that do not fully reflect what the actors themselves would consider most relevant to measure. Relatedly, we applied equal weighting across all interviewees, indicators, and subcategories in the absence of a stakeholder-informed or otherwise justified alternative weighting scheme. Validation interviews that could have informed a weighting scheme were only conducted in Brazil with a small sample and not in the DRC, where the escalation of conflict in the study region prevented additional fieldwork. This further limited the feasibility of deriving a robust, cross-country weighting approach. Future S-LCA studies could strengthen contextual validity by involving actors already at the subcategory and indicator selection stage or by exploring stakeholder-informed weighting approaches.

## Conclusion and outlook

This study contributes to the limited body of S-LCA research on coffee production, and, to our knowledge, is among the first to be based on primary data collected in Brazil and the DRC. It also appears to be among the first S-LCA studies to compare the responses of different interviewee groups and aggregate the responses of different interviewees rather than simply grading social performance based on different data sources. With this study, we could show that regional context and organization influence the social performance of our coffee case studies. Our findings are based on a purposively selected sample in two specific regions and should be understood as reflective of these case studies, rather than as generalizable claims about coffee production more broadly. Overall, the assessed case studies showed higher social performance than reported in previous literature, likely due to the strong presence of certified cooperatives in our sample, whereas most literature addresses general global impacts and reflects the overall situation. However, to truly transform the coffee sector, the case studies would need to achieve scores between 1 and 2 (beyond compliance), since mere compliance with laws and regulations does not yet ensure decent livelihoods for all actors involved, as demonstrated by minimum wages that are insufficient to meet basic needs.

To further develop and standardize the S-LCA methodology, and the reference scale approach in particular, creating a database with country or even region- and sector-specific reference scales rather than social impacts or risks could be a way forward. Such a database could then serve as a basis for social performance assessment of specific companies or organizations and ensure their comparability. This would give practitioners a concrete reference in each country against which they can compare site-specific results. Such an effort would be very labor- and knowledge-intensive but could contribute to meaningful applicability of the method. With AI-aided approaches, this might become more feasible in the near future. Ultimately, much more research is needed to achieve greater case sensitivity in S-LCA before such assessments become mandatory or policy decisions are based on them.

## Supplementary Information

Below is the link to the electronic supplementary material.

Supplementary Material 1: Online Resource 1: General supplementary information.

Supplementary Material 2: Online Resource 2: Reference scales.

Supplementary Material 3: Online Resource 3: Interview guidelines: example of producers in Brazil.

## Data Availability

The dataset used in this study can be found in the figshare repository: https://figshare.com/s/a5199903a575cc191e20 (as soon as the review process is over, a DOI will be created).

## Footnotes

1 *Metayers* are a type of sharecropper who are allowed to grow crops on someone else’s land in exchange for 1–2 days of free labor per week.

2 Brazilian Real.

3 The RPA (Recibo de Pagamento Autônomo) contract is a self-employed payment receipt used in Brazil for independent workers who do not have a formal employment contract under the CLT (Consolidation of Labor Laws).

4 The Carteira Assinada is the formal employment contract in Brazil. It ensures labor rights, social security, paid holidays, and severance benefits.

5 Outubro Rosa (Pink October) is a Brazilian public health campaign focused on breast cancer awareness and prevention through screenings.

6 Novembro Azul (Blue November) is a similar campaign focused on men’s health, particularly prostate cancer awareness and screenings.

7 SENAR (Serviço Nacional de Aprendizagem Rural) is a Brazilian organization that offers educational programs, training, and technical support to agricultural workers and producers.

8 Association Villageoise d’Epargne et de Crédit.

9 Promotion et Appui aux Initiatives de Développement Économique du Kivu.

10 Congolese Franc.

## References

[CR1] Anker R (2006) Living wages around the world: A new methodology and internationally comparable estimates. ILR 145:309–338. 10.1111/j.1564-913X.2006.tb00037.x

[CR2] Belandi C (2019) Blacks and browns are the minority heading large agricultural establishments | News Agency. In: Agência de Notícias - IBGE. https://agenciadenoticias.ibge.gov.br/en/agencia-news/2184-news-agency/news/26175-blacks-and-browns-are-the-minority-heading-large-agricultural-establishments. Accessed 2 Feb 2026

[CR3] Benoit-Norris C, Cavan DA, Norris G (2012) Identifying Social Impacts in Product Supply Chains:Overview and Application of the Social Hotspot Database. Sustain 4:1946–1965. 10.3390/su4091946

[CR4] Bizikova L, Nkonya E, Minah M et al (2020) A scoping review of the contributions of farmers’ organizations to smallholder agriculture. Nat Food 1:620–630. 10.1038/s43016-020-00164-x 37128116

[CR5] Borrella I, Mataix C, Carrasco-Gallego R (2015) Smallholder Farmers in the Speciality Coffee Industry: Opportunities, Constraints and the Businesses that are Making it Possible. IDS Bull 46:29–44. 10.1111/1759-5436.12142

[CR6] Bray DB, Sánchez JLP, Murphy EC (2002) Social Dimensions of Organic Coffee Production in Mexico: Lessons for Eco-Labeling Initiatives. SNR 15:429–446. 10.1080/08941920252866783

[CR7] Brenes-Peralta L, Jiménez-Morales MF, Campos-Rodríguez R, Vittuari M (2021) Unveiling the social performance of selected agri-food chains in Costa Rica: the case of green coffee, raw milk and leafy vegetables. Int J Life Cycle Assess 26:2056–2071. 10.1007/s11367-021-01964-4 PMC8428208 34522069

[CR8] Cellura M, De Luca AI, Iofrida N, Mistretta M (2024) Social Life Cycle Assessment of Batteries. In: Passerini S, Barelli L, Baumann M et al (eds) Emerging Battery Technologies to Boost the Clean Energy Transition. Springer International Publishing, Cham, pp 291–306

[CR9] Chabrawi AMRO, De Andrade JM, Ugaya CML, Traverso M (2024) Towards reliable primary data collection and harmonized set of indicators in S-LCA on the stakeholder worker. Int J Life Cycle Assess. 10.1007/s11367-024-02400-z

[CR10] Cordes KY, Sagan M, Kennedy S (2021) Responsible Coffee Sourcing: Towards a Living Income for Producers. Columbia Center on Sustainable Investment

[CR44] R Core Team (2024) _R: A Language and Environment for Statistical Computing_

[CR11] Crenshaw KW (1994) Mapping the Margins: Intersectionality, Identity Politics, and Violence Against Women of Color. In: The Public Nature of Private Violence, 1st Edition. Routledge, pp 93–118

[CR12] Dedieu B, Contzen S, Nettle R et al (2022) The Multiple Influences on the Future of Work in Agriculture: Global Perspectives. Front Sustain Food Syst 6:889508. 10.3389/fsufs.2022.889508

[CR13] Díez-Hernández J, Carreira-Barral I, López-de-Foronda Ó, Martel-Martín S (2026) Database-assisted Social Life Cycle Assessment for innovations: Lessons learnt from application. Environ Impact Assess Rev 116:108151. 10.1016/j.eiar.2025.108151

[CR14] Dolce J (2020) Popular agrarian reform and the struggle for land in Brazil. Institute for Social Research, Tricontinental

[CR15] Elsby A (2020) Creaming off commodity profits: Europe’s re-export boom and Africa’s earnings crisis in the coffee and cocoa sectors. ROAPE 47:638–650. 10.1080/03056244.2020.1842186

[CR16] FAO (2025) Coffee. https://www.fao.org/markets-and-trade/commodities-overview/beverages/coffee/en. Accessed 12 Feb 2025

[CR17] Fernandes BM (2013) Re-peasantization, Resistance and Subordination: The Struggle for Land and Agrarian Reform in Brazil. Agrar S 2:269–289. 10.1177/2277976013517200

[CR18] Ferrante L, Vilani RM, Apurinã W et al (2025) Brazil’s Indigenous lands face legal setbacks. Science 389:465–465. 10.1126/science.ady1746 40743355

[CR19] Franck A, Prapha A (2021) Not in This Together: How supermarkets became pandemic winners while women workers are losing out. Oxfam

[CR20] Grabs J, Ponte S (2019) The evolution of power in the global coffee value chain and production network. J Econ Geogr 19:803–828. 10.1093/jeg/lbz008

[CR21] Grant T (2021) Gender equity in the Democratic Republic of Congo’s coffee sector. In: Perfect Daily Grind. https://perfectdailygrind.com/2021/04/gender-equity-in-the-democratic-republic-of-congos-coffee-sector/. Accessed 2 Feb 2026

[CR22] Harmens R, Pillay S, Davis D et al (2022) Product Social Impact Assessment- Social Topics Report. Amersfoort

[CR23] Hjerl Hansen J, Christensen AB, Andersen Thing D, Sjöström T (2016) BITTER Coffee, Slavery-like Working Conditions and deadly Pesticides on Brazilian Coffee Plantations. DANWATCH

[CR24] Hodgetts DJ, Young-Hauser AM, Arrowsmith J et al (2022) Deliberating Upon the Living Wage to Alleviate In-Work Poverty: A Rhetorical Inquiry Into Key Stakeholder Accounts. Front Psychol 13:810870. 10.3389/fpsyg.2022.810870 PMC9204092 35719495

[CR25] International Alert (2025) Protecting Indigenous peoples’ rights and nature in eastern DRC: the role of peacebuilding. https://www.international-alert.org/stories/protecting-indigenous-peoples-rights-and-nature-in-eastern-drc/. Accessed 2 Feb 2026

[CR26] ISO (2024) ISO 14075 Environmental management - principle and framework for social life cycle assessment

[CR27] Lucchetti MC, Arcese G, Traverso M, Montauti C (2018) S-LCA applications: a case studies analysis. E3S Web Conf 74:10009. 10.1051/e3sconf/20187410009

[CR28] Luna Ostos LM, Roche L, Coroama V, Finkbeiner M (2024) Social life cycle assessment in the chocolate industry: A Colombian case study with Luker Chocolate. Int J Life Cycle Assess 29:929–951. 10.1007/s11367-023-02261-y

[CR29] Maguire-Rajpaul VA, Rajpaul VM, McDermott CL, Guedes Pinto LF (2020) Coffee certification in Brazil: compliance with social standards and its implications for social equity. Environ Dev Sustain 22:2015–2044. 10.1007/s10668-018-0275-z

[CR30] Medeiros Guimarães Y, Pires Eustachio JHP, Leal Filho W et al (2022) Drivers and barriers in sustainable supply chains: The case of the Brazilian coffee industry. SCP 34:42–54. 10.1016/j.spc.2022.08.031

[CR31] Medinaceli A, Andersen LE, Delajara M et al (2023) Living Wage Update Report: Minas Gerais South/Southwestern Region, Brazil, 2023. Anker Reserach Institute, Global Living Wage Coalition, SDSN Bolivia

[CR33] Meemken E-M, Sellare J, Kouame CN, Qaim M (2019) Effects of Fairtrade on the livelihoods of poor rural workers. Nat Sustain 2:635–642. 10.1038/s41893-019-0311-5

[CR32] Meemken E-M, Barrett CB, Michelson HC et al (2021) Sustainability standards in global agrifood supply chains. Nat Food 2:758–765. 10.1038/s43016-021-00360-3 37117971

[CR34] Ministério do Trabalho e Emprego (2025) Cadastro de Empregadores que tenham submetido trabalhadores a condições análogas à de escravo, Portaria Interministerial MTE/MDHC/MIR n^o^ 18 de 13/09/2024. Brazil

[CR35] Morais MO, da César P (2021) Political architectures in the municipality of Varre-Sai (Brazil): for *sustainabilities* in ‘specialty coffees’ production management. R Soc Open Sci 8:201874. 10.1098/rsos.201874 PMC8074927 33959346

[CR36] Musoko P (2022) Comment: Achieving long-term food security in DRC is all about building resilience World Food Programme. https://www.wfp.org/stories/comment-achieving-long-term-food-security-drc-all-about-building-resilience. Accessed 13 Jan 2026

[CR37] Nakaharada CM, Eduardo C, Drummond Pinheiro F et al (2022) Workers’ Rights and Social Protection In Brazil Legal and Policy Gaps. OECD Watch and Conectas Human Rights

[CR38] Palinkas LA, Horwitz SM, Green CA et al (2015) Purposeful Sampling for Qualitative Data Collection and Analysis in Mixed Method Implementation Research. Adm Policy Ment Health 42:533–544. 10.1007/s10488-013-0528-y PMC4012002 24193818

[CR39] Panhuysen S, de Vries F (2023) Coffee Barometer 2023

[CR40] Panhuysen S, Pierrot J (2020) Coffee Barometer 2020

[CR41] Pereira MI, Ferreira EB (2022) Café feminino: mulheres organizadas buscando igualdade no Sul de Minas. REVER 11:70–94

[CR42] Perosa BB, Saes MSM, Jesus CMD (2024) Between law and voluntary sustainability standards: a case study of the labor conditions in Brazilian coffee production. Rev Econ Sociol Rural 62:e275418. 10.1590/1806-9479.2023.275418

[CR43] Pollok L, Spierling S, Endres H-J, Grote U (2021) Social Life Cycle Assessments: A Review on Past Development, Advances and Methodological Challenges. Sustain 13:10286. 10.3390/su131810286

[CR45] Rahmah DM, Purnomo D, Filianty F et al (2023) Social Life Cycle Assessment of a Coffee Production Management System in a Rural Area: A Regional Evaluation of the Coffee Industry in West Java. Indonesia Sustain 15:13834. 10.3390/su151813834

[CR46] Ramirez PKS, Petti L, Haberland NT, Ugaya CML (2014) Subcategory assessment method for social life cycle assessment. Part 1: methodological framework. Int J Life Cycle Assess 19:1515–1523. 10.1007/s11367-014-0761-y

[CR47] Raposeiras I, Couto C (2021) O livro da barista - instrumentos teóricos para um bom profissional do café. Coffee Lab, São Paulo

[CR48] Rehman M, Petrillo A, De Felice F (2025) Environmental, Social, and Economic Life Cycle Assessment of the Italian Coffee Supply Chain. Cureus j eng. 10.7759/s44388-025-05869-y

[CR49] Richey LA, Ponte S (2021) Brand Aid and coffee value chain development interventions: Is Starbucks working aid out of business? World Dev 143:105193. 10.1016/j.worlddev.2020.105193

[CR50] Roche L, Holzapfel P, Finkbeiner M (2025) Primary data share indicator for social life cycle assessment. J Clean Prod 497:145051. 10.1016/j.jclepro.2025.145051

[CR51] Rodney W (1972) How Europe Underdeveloped Africa. Bogle-L’Ouverture Publications & Tanzanian Publishing House, London

[CR52] Sachs J, Cordes KY, Rising J et al (2019) Ensuring Economic Viability and Sustainability of Coffee Production. Columbia Center on Sustainable Investment

[CR53] Santos VP, Ribeiro PCC, Rodrigues LB (2022) Sustainability assessment of coffee production in Brazil. Environ Sci Pollut Res. 10.1007/s11356-022-22922-z 36094709

[CR54] Sehlin MacNeil K, Daniels-Mayes S, Akbar S et al (2021) Social Life Cycle Assessment Used in Indigenous Contexts: A Critical Analysis. Sustain 13:5158. 10.3390/su13095158

[CR55] Sigcha E, Sucozhañay D, Cabrera F et al (2024) Applying social life cycle assessment in the informal recycling sector: Understanding challenges and limitations. Waste Manag 181:20–33. 10.1016/j.wasman.2024.03.029 38574689

[CR56] Sigrist M, Thom B, Gabriela S et al (2025) Coffee Work Outside Plantations - Emancipation, Fame and Limits for the Next Generation of Coffee Growers in Minas Gerais (Brazil). Anthropol Soc 49:193–216. 10.7202/1119573ar

[CR57] Sivaraman A (2020) Five Things You Need to Know About Social Sustainability and Inclusion. In: World Bank. https://www.worldbank.org/en/news/feature/2020/09/02/five-things-about-social-sustainability-and-inclusion. Accessed 13 Jan 2026

[CR58] Slosse W, Buysse J, Vijaya V et al (2023) Coffee cooperatives and cross-border side-sales in the Eastern Democratic Republic of Congo: two sides of the same coin. Int Food Agribus Manag Rev 26:691–709. 10.22434/ifamr2022.0131

[CR60] Thom B, Sigrist M, Späth L et al (2025) Mismatches in the coffee value chain: from basic needs to hedonistic lifestyles—experiences from Brazil, the Democratic Republic of Congo, and Switzerland. Front Sustain Food Syst 9:1623366. 10.3389/fsufs.2025.1623366

[CR59] Thom B, Pfister S, Malembaka RB et al (2026) Beyond the average: understanding the heterogeneity of environmental impacts of green coffee in Brazil and the Democratic Republic of the Congo. J Clean Prod 546:147805. 10.1016/j.jclepro.2026.147805

[CR61] Tokede O, Traverso M (2020) Implementing the guidelines for social life cycle assessment: past, present, and future. Int J Life Cycle Assess 25:1910–1929. 10.1007/s11367-020-01814-9

[CR62] Traverso M, Mankaa RN (2025) The social life cycle assessment of products and organizations—methodological developments and implementations. Int J Life Cycle Assess 30:1011–1017. 10.1007/s11367-025-02480-5

[CR63] Traverso M, Petti L, D’Eusanio M et al (2025) Finally, an international standard on social life cycle assessment: ISO 14075. Int J Life Cycle Assess. 10.1007/s11367-025-02522-y. s11367-025-02522-y

[CR64] UNDP (2025) Climate Resilient Growth and Adaptation in the Democratic Republic of the Congo | UNDP Climate Change Adaptation. https://www.adaptation-undp.org/projects/climate-resilient-growth-and-adaptation-democratic-republic-congo. Accessed 13 Jan 2026

[CR65] UNEP (2020) Guidelines for Social Life Cycle Assessment of Products and Organizations 2020. United Nations Environment Programme (UNEP)

[CR66] UNEP (2021) Methodological Sheets for Subcategories in Social Life Cycle Assessment. United Nations Environment Programme (UNEP)

[CR67] Vinci G, Prencipe SA, Ruggeri M et al (2025) Sustainability performance evaluation in the organic durum wheat production: evidence from Italy. Int J Life Cycle Assess 30:1115–1133. 10.1007/s11367-024-02302-0

[CR68] Zimdars C, Haas A, Pfister S (2018) Enhancing comprehensive measurement of social impacts in S-LCA by including environmental and economic aspects. Int J Life Cycle Assess 23:133–146. 10.1007/s11367-017-1305-z

