# Supplementary material for: Towards greater sensitivity: advancing social life cycle assessment for the coffee sector in Brazil and the Democratic Republic of the Congo
Source: Int J Life Cycle Assess. 2026 Oct 18;31(11):190. doi: 10.1007/s11367-026-02760-8 (PMC13677624; doi:10.1007/s11367-026-02760-8)
Supplement: Supplementary file 1 [file 11367_2026_2760_MOESM1_ESM.pdf]

Online Resource 1

Towards greater sensitivity: advancing social life cycle assessment for the coffee sector in Brazil and the Democratic Republic of the Congo

The International Journal of Life Cycle Assessment

Authors: Braida Thom[1], Lea Fabritius[1], Marie Sigrist[1], Didace Banyanga[2], Stephan Pfister[3], Réussite Bugale Malembaka[1], Gabriela Schneider[4], Kokou Kintche[2], Johanna Jacobi[1]

[1]Agroecological Transitions, Department of Environmental Systems Sciences, ETH Zurich, Switzerland, Universitätstrasse 2, 8092 Zurich, Switzerland
[2] International Institute of Tropical Agriculture (IITA), Route Kavumu, Km 18, Bifurcation Birava, Site Kalambo, Bukavu, Democratic Republic Congo
[3] Ecological Systems Design, Department of Civil, Environmental and Geomatic Engineering, ETH Zurich, Switzerland, Laura-Hezner-Weg 7, 8093 Zurich, Switzerland
[4] Programa de Ciencias Sociais em Desenvolvimento, Agricultura e Sociedade, Universidade Federal Rural do Rio de Janeiro (UFRRJ), BR-465, Km 7 - Seropédica, Rio de Janeiro, Brazil

Correspoding author: Braida Thom (braida.thom@gmail.com)

Table of Contents

S1 Tables 2
S2 Contextual factors 7
S3 Figures 9
S4 References 12

S1 Tables

Table SI 1: Sociodemographic data and main functions of workers.

Case study			B1: direct trade cooperative	B2: women's group	B3: social movement	B4: alternative specialty coffee plantation	B5: conventional specialty coffee plantation	D1: metayage plantation	D2: female-led cooperative	D3: established cooperative	D4: emerging cooperative
WORKERS	Gender	female (n)	2	3	2	4	4	4	4	2	2
		male	7	7	4	6	7	5	4	7	6
		other									
	Age		Mean 30.9; std dev 12.1	Mean 33.9; std dev 9.4	Mean 38.3; std dev 14.3	Mean 35; std dev 14.1	Mean 41.7; std dev 11.2	Mean 35; std dev 15.1	Mean 40.5; std dev 14.2	Mean 34.9; std dev 12.5	Mean 30.9; std dev 10.2
	Ethnicity[a]	white (n)	6	6	1	2	3				
		brown	3	3	4	7	4				
		black		1		1	4				
		Indigenous			1						
		Shi						9	8		
		Havu								9	8
	Main functions		2 rural/farm workers (p[b])<br>2 tractor drivers and rural/farm worker (p)<br>1 coffee grader<br>1 coffee grader and roasting assistant(p)<br>1 coffee roaster (p)<br>1 finance and operations manager (p)<br>1 accountant (p)	1 rural/farm worker (d[b])<br>1 agricultural machinery operator and coordinator (p)<br>1 warehouse coordinator (p)<br>1 warehouse machinery operator (p)<br>1 warehouse worker (p)<br>1 general services worker (p)<br>1 administrative and environmental assistant (p)<br>1 coffee roasting supervisor (p)<br>1 sales representative (p)<br>1 sales supervisor (p)	1 rural/farm worker (p)<br>2 nursery workers (p)<br>1 warehouse and logistics assistant (p)<br>1 administrative manager (p)<br>1 commercial manager (p)	3 rural/farm workers (p)<br>2 tractor drivers (p)<br>1 gardener (p)<br>1 coffee processor and grader (p)<br>1 coffee mill operator and grader (p)<br>1 coffee grader (p)<br>1 administrative assistant (p)	3 rural/farm workers (p)<br>2 tractor drivers (p)<br>1 tractor driver and rural/farm worker (p)<br>1 mechanic and driver (p)<br>1 general services worker (p)<br>1 coffee cupper (p)<br>1 cleaner and roasting assistant (p)<br>1 coffee packer and grinder (p)	1 rural/farm worker (d)<br>1 security guard (p)<br>1 washing station machinery operator (s)<br>1 coffee sorter (d)<br>1 coffee sorter (s)<br>1 cashier and coffee sorter (s)<br>1 coffee grader (s)<br>1 day labor and metayer supervisor (p)<br>1 accountant (s)	1 washing station worker (d)<br>1 coffee sorter and drying operator (d)<br>2 coffee sorters (d)<br>1 washing station machinery operator (s[b])<br>1 cashier (s)<br>1 agronomist (s)<br>1 cooperative manager (p)	1 cherry collector (s)<br>1 cherry buyer (s)<br>1 collection point supervisor (s)<br>2 washing station worker (s)<br>1 coffee sorter (s)<br>1 washing station quality manager (s)<br>1 coffee cupper (p)<br>1 agronomist (p)	1 cherry collector and coffee sorter (d)<br>1 coffee sorter (s)<br>1 washing station machinery operator (s)<br>1 washing station quality manager (s)<br>1 washing station supervisor (s)<br>1 washing station coordinator (s)<br>1 cashier (p)<br>1 cooperative manager (p)

	Wage (< min)[c]	white (n)									
		brown									
		black									
		Indigenous									
		Shi						7 (78%)	5 (63%)		
		Havu								6 (67%)	6 (75%)
		male						5 (100%)	2 (50%)	4 (57%)	5 (83%)
		female						2 (50%)	3 (75%)	2 (100%)	1 (50%)
	Wage (= min)[d]	white (n)	2 (33%)			1 (50%)					
		brown	2 (67%)		3 (75%)	3 (43%)	2 (50%)				
		black					1 (25%)				
		Indigenous			1 (100%)						
		Shi									
		Havu									
		male	2 (29%)		4 (100%)	2 (33%)	1 (14%)				
		female	2 (100%)			2 (50%)	2 (50%)				
	Wage (> min)[e]	white (n)	4 (67%)	6 (100%)	1 (100%)	1 (50%)	3 (100%)				
		brown	1 (33%)	3 (100%)	1 (25%)	4 (57%)	2 (50%)				
		black		1 (100%)		1 (100%)	3 (75%)				
		Indigenous									
		Shi						2 (22%)	3 (38%)		
		Havu								3 (33%)	2 (25%)
		male	5 (71%)	7 (100%)		4 (66%)	6 (85%)		2 (50%)	3 (43%)	1 (17%)
		female		3 (100%)	2 (100%)	2 (50%)	2 (50%)	2 (50%)	1 (25%)		1 (50%)

[a] In Brazil, the categories were taken from the IBGE Brazilian census 2022, in the DRC, the question was asked as open question.
[b] p= permanent worker; s= seasonal worker; d= day laborer
[c] Number of people earning a salary below the minimum wage
[d] Number of people earning a minimum wage
[e] Number of people earning a salary above the minimum wage

Table SI 2: Sociodemographic data for metayers, farmers, and management, and main functions of the management group.

Case study			B1: direct trade cooperative	B2: women's group	B3: social movement	B4: alternative specialty coffee plantation	B5: conventional specialty coffee plantation	D1: metayage plantation	D2: female-led cooperative	D3: established cooperative	D4: emerging cooperative
FARMERS/METAYERS	Gender	female	3	10	1			5	10	5	3
FARMERS/METAYERS	Gender	male	7		9			5		5	7
FARMERS/METAYERS	Gender	other									
FARMERS/METAYERS	Age		Mean 49.6<br>std dev 11.9	Mean 49.4<br>std dev 7	Mean 50.1<br>std dev 10.6	Mean<br>std dev	Mean<br>std dev	Mean 51.7<br>std dev 13.5	Mean 54<br>std dev 13.1	Mean 50.5<br>std dev 18.7	Mean 47.8<br>std dev 14.2
FARMERS/METAYERS	Ethnicity[a]	white	9	9	5						
FARMERS/METAYERS	Ethnicity[a]	brown	1	1	4						
FARMERS/METAYERS	Ethnicity[a]	black			1						
FARMERS/METAYERS	Ethnicity[a]	Shi						8	10		
FARMERS/METAYERS	Ethnicity[a]	Havu								10	10
FARMERS/METAYERS	Ethnicity[a]	NA						2			
MANAGEMENT	Gender	female		1		1	1				
MANAGEMENT	Gender	male	1		1			5	3	4	3
MANAGEMENT	Gender	other									
MANAGEMENT	Age		Mean 44<br>std dev -	Mean 32<br>std dev -	Mean 52<br>std dev	Mean 59<br>std dev -	Mean 60<br>std dev	Mean NA<br>std dev -	Mean NA<br>std dev -	Mean NA<br>std dev -	Mean NA<br>std dev -
MANAGEMENT	Ethnicity	white	1	1		1	1				
MANAGEMENT	Ethnicity	brown									
MANAGEMENT	Ethnicity	black			1						
MANAGEMENT	Ethnicity	NA						5	3	4	3
MANAGEMENT	Main functions		1 cooperative president	1 social and environmental director	1 cooperative president	1 owner	1 owner	1 washing station supervisor and agronomist<br>2 agronomists<br>1 agronomist and company manager<br>1 business advisor	2 washing station supervisors<br>1 cooperative president	2 washing station supervisors<br>1 president of washing stations<br>1 cooperative president	2 washing station supervisors<br>1 cooperative president

[a] In Brazil, the categories were taken from the IBGE Brazilian census 2022, in the DRC, the question was asked as open question.

Table SI 3: Kruskal-Wallis test and post hoc Dunn's test with Bonferroni correction: comparison of the interviewee group workers across all impact subcategories of the stakeholder categories workers, value chain actors, and local community, and across case studies.

Impact subcategory	chi square	DF	p-value	significant pairs	N sig.pairs
Freedom of association and collective bargaining	25.786	9	0.0022**	B2_W > D1_M[a]; B3_W > D1_M	2
Child labor (w)	16.18	9	0.0632	-	0
Fair salary	50.259	9	<0.0001***	B1_W > D1_M; B2_W > D1_M; B4_W > D1_M; B5_W > D1_M; B2_W > D1_W; B2_W > D2_W; B2_W > D3_W	7
Working hours	33.584	9	0.0001***	B2_W < D1_M; D1_M > D1_W; D1_M > D3_W; D1_M > D4_W	4
Equal opportunities/discrimination (w)	7.124	4	0.1295	-	0
Health and safety (w)	69.899	9	<0.0001***	B1_W > D1_M; B2_W > D1_M; B4_W > D1_M; B5_W > D1_M; B2_W > D1_W; B1_W > D2_W; B2_W > D2_W; B5_W > D2_W; B1_W > D3_W; B2_W > D3_W; B4_W > D3_W; B5_W > D3_W; B1_W > D4_W; B2_W > D4_W; B5_W > D4_W	15
Social benefits/social security (w)	45.429	9	<0.0001***	B2_W > B5_W; B1_W > D1_M; B2_W > D1_M; B4_W > D1_M; D1_M < D1_W; B2_W > D3_W	6
Employment relationship	64.405	9	<0.0001***	B1_W > D1_M; B2_W > D1_M; B4_W > D1_M; B5_W > D1_M; B1_W > D1_W; B2_W > D1_W; B5_W > D1_W; B1_W > D2_W; B2_W > D2_W; B5_W > D2_W; B1_W > D3_W; B2_W > D3_W; B4_W > D3_W; B5_W > D3_W	14
Livelihood and well-being (w)	49.32	9	<0.0001***	B1_W > D1_M; B2_W > D1_M; B3_W > D1_M; B1_W > D3_W; B2_W > D3_W; B1_W > D4_W; B2_W > D4_W; B3_W > D4_W	8
Promoting social responsibility	29.089	9	0.0006***	-	0
Supplier relationships	NA	NA	NA	-	0
Wealth distribution	NA	NA	NA	-	0
Access to material resources	36.608	9	<0.0001***	B2_W < D4_W; B4_W < D4_W; B5_W < D4_W; D1_M < D4_W; D1_W < D4_W; D2_W < D4_W; D3_W < D4_W	7
Access to immaterial resources	40.57	9	<0.0001***	B2_W > B5_W; B2_W > D1_M; B2_W > D2_W; D1_M < D4_W	4
Cultural heritage	32.224	9	0.0002***	B2_W > B5_W; B2_W > D1_M; B2_W > D1_W; B2_W > D2_W	4
Safe and healthy working conditions	20.063	9	0.0175*	-	0
Community engagement	21.301	9	0.0114*	-	0
Local employment	11.97	9	0.2150	-	0
Mean across all impact subcategories	67.236	9	<0.0001***	B1_W > D1_M; B2_W > D1_M; B3_W > D1_M; B4_W > D1_M; B5_W > D1_M; B2_W > D1_W; B1_W > D2_W; B2_W > D2_W; B1_W > D3_W; B2_W > D3_W; B3_W > D3_W; B4_W > D3_W; B2_W > D4_W	13

[a] W stays for worker, M stays for Metayer; *** p-value < 0.001; ** p-value < 0.01; * p-value < 0.05

Table SI 4: Kruskal-Wallis test and post hoc Dunn's test with Bonferroni correction: comparison of the interviewee group farmers across all impact subcategories of the stakeholder categories farmers, value chain actors, and local community, and across case studies.

Impact subcategory	chi square	DF	p-value	significant pairs	N sig. pairs
Livelihood and well-being (f)	41.903	5	<0.0001***	B1 > D2; B2 > D2; B1 > D3; B2 > D3; B3 > D3; B1 > D4; B2 > D4; B3 > D4	8
Inclusiveness	28.72	5	<0.0001***	B2 > B3; B2 > D2; B2 > D4	3
Productivity	10.871	5	0.0540		0
Access to services	30.893	5	<0.0001***	B1 > D2; B2 > D2; B1 > D3; B2 > D3; B1 > D4	5
Trading relationships	48.714	5	<0.0001***	B1 > D2; B2 > D2; B3 > D2; B1 > D3; B2 > D3; B3 > D3; B1 > D4; B2 > D4; B3 > D4	9
Next generation farmers	28.297	5	<0.0001***	B1 < B2; B2 > B3; B2 > D2; B2 > D3; B2 > D4	5

Equal opportunities/discrimination (f)	17.466	2	0.0002***	B1 < B2; B2 > B3	2
Social benefits/social security (f)	23.216	5	0.0003***	B1 < B2; B2 > B3; B2 > D2; B2 > D3	4
Health and safety (f)	49.387	5	<0.0001***	B1 > D2; B2 > D2; B3 > D2; B1 > D3; B2 > D3; B1 > D4; B2 > D4	7
Child labor (f)	7.246	5	0.2030		0
Promoting social responsibility	38.144	5	<0.0001***	B1 > B3; B2 > B3; B1 > D2; B2 > D2; B1 > D3; B2 > D3; B1 > D4; B2 > D4	8
Supplier relationships	29.301	5	<0.0001***	B1 < B2; B2 > B3; B2 > D2; B2 > D3; B2 > D4	5
Wealth distribution	31.004	5	<0.0001***	B1 > D2; B2 > D2; B3 > D2; B1 > D4; B2 > D4; B3 > D4	6
Access to material resources	19.728	5	0.0014**	B1 > D2; B1 > D3	2
Access to immaterial resources	23.663	5	0.0003***	B2 > B3; B2 > D3	2
Cultural heritage	33.845	5	<0.0001***	B2 > B3; B2 > D2; B2 > D3; B2 > D4	4
Safe and healthy working conditions	42.503	5	<0.0001***	B1 > B3; B2 > B3; B1 > D2; B2 > D2; B1 > D3; B2 > D3; B1 > D4; B2 > D4	8
Community engagement	29.507	5	<0.0001***	B2 > B3; B1 > D2; B2 > D2; B1 > D3; B2 > D3	5
Local employment	9.983	5	0.0757		0
Mean across all impact subcategories	51.809	5	<0.0001***	B1 > D2; B2 > D2; B3 > D2; B1 > D3; B2 > D3; B2 > D4	6

*** p-value < 0.001; ** p-value < 0.01; * p-value < 0.05

Table SI 5: Comparison of the interviewee group management across all impact subcategories of the stakeholder categories workers, farmers, value chain actors, and local community, and across case studies. The table shows pairs with large differences in mean performance scores (≥1.0).

Impact subcategory	Pairs with large differences (≥1.0)	N pairs
Freedom of association and collective bargaining	B2 > D1	1
Child labor (w)	-	-
Fair salary	B2 > D1; B2 > D2; B2 > D3; B2 > D4; B3 > D1; B3 > D2; B3 > D3; B3 > D4; B4 > D1; B4 > D2; B4 > D3; B4 > D4	12
Working hours	-	-
Equal opportunities/discrimination (w)	B1 > B5; B1 > D1; B2 > B1; B2 > B3; B2 > B4; B2 > B5; B2 > D1; B2 > D2; B2 > D3; B2 > D4; B3 > B5; B3 > D1; B4 > B5; B4 > D1; D2 > B5; D2 > D1; D3 > B5; D3 > D1; D4 > B5; D4 > D1	20
Health and safety (w)	B1 > D1; B1 > D2; B1 > D3; B1 > D4; B2 > D1; B2 > D2; B2 > D3; B2 > D4; B3 > D1; B3 > D3; B3 > D4; B4 > D1; B4 > D3; B4 > D4; B5 > D1; B5 > D2; B5 > D3; B5 > D4	18
Social benefits/social security (w)	B1 > B5; B1 > D4; B2 > B3; B2 > B4; B2 > B5; B2 > D1; B2 > D2; B2 > D3; B2 > D4	9
Employment relationship	B2 > B1; B2 > B3; B4 > B1; B4 > B3; B5 > B1; B5 > B3	6
Trading relationships	-	-
Next generation farmers	B2 > B1; B2 > B3; B2 > D2; B2 > D3; B2 > D4	5
Equal opportunities/discrimination (f)	B2 > B1; B2 > B3; B2 > D2; B2 > D3; B2 > D4	5
Social benefits/social security (f)	B1 > B3; B1 > D4; B2 > B1; B2 > B3; B2 > D2; B2 > D3; B2 > D4; D2 > B3; D2 > D4; D3 > B3; D3 > D4	11
Health and safety (f)	B1 > D2; B1 > D3; B1 > D4; B2 > D2; B2 > D3; B2 > D4; B3 > D3; B3 > D4	8
Promoting social responsibility	B1 > B3; B1 > B4; B1 > B5; B1 > D1; B1 > D2; B1 > D3; B1 > D4; B2 > B3; B2 > B4; B2 > B5; B2 > D1; B2 > D2; B2 > D3; B2 > D4	14
Supplier relationships	B2 > B1; B2 > B3; B2 > B4; B2 > B5; B2 > D1; B2 > D2; B2 > D3; B2 > D4; B5 > B1; B5 > B3; B5 > B4; B5 > D1; B5 > D2; B5 > D3; B5 > D4	15
Wealth distribution	B2 > B1; B2 > B4; B2 > B5; B2 > D1; B2 > D2; B2 > D3; B2 > D4; B3 > B1; B3 > B4; B3 > B5; B3 > D1; B3 > D2; B3 > D3; B3 > D4	14
Access to material resources	-	-
Access to immaterial resources	B1 > D1; B1 > D2; B1 > D4; B2 > B1; B2 > B3; B2 > B4; B2 > B5; B2 > D1; B2 > D2; B2 > D3; B2 > D4; B3 > D1; B3 > D2; B3 > D4; B4 > D1; B4 > D2; B4 > D4; B5 > D1; B5 > D2; B5 > D4; D3 > D1; D3 > D2; D3 > D4	23
Cultural heritage	B1 > B3; B1 > D1; B1 > D2; B1 > D4; B2 > B1; B2 > B3; B2 > B4; B2 > B5; B2 > D1; B2 > D2; B2 > D3; B2 > D4; B4 > B3; B4 > D1; B4 > D2; B4 > D4; B5 > B3; B5 > D1; B5 > D2; B5 > D4; D3 > B3; D3 > D1; D3 > D2; D3 > D4	24

Safe and healthy working conditions	B1 > B3; B1 > B4; B1 > B5; B1 > D1; B1 > D2; B2 > B3; B2 > B4; B2 > B5; B2 > D1; B2 > D2; D3 > B3; D3 > B4; D3 > B5; D3 > D1; D3 > D2; D4 > B3; D4 > B4; D4 > B5; D4 > D1; D4 > D2	20
Community engagement	B1 > B4; B1 > B5; B1 > D1; B1 > D3; B1 > D4; B2 > B4; B2 > B5; B2 > D1; B2 > D3; B2 > D4; B3 > B4; B3 > B5; B3 > D1; B3 > D3; B3 > D4; D2 > B4; D2 > B5; D2 > D1; D2 > D3; D2 > D4	20
Local employment	-	-
Mean across all impact subcategories	B2 > D1	1

Table SI 6: Comparison of interviewee groups within each case study across all impact subcategories of the stakeholder categories workers, farmers, value chain actors, and local community. The table shows pairs with large differences in mean performance scores (≥1.0).

Impact subcategory	Pairs with large differences (≥1.0)	N pairs
Freedom of association and collective bargaining	-	-
Child labor (w)	-	-
Fair salary	-	-
Working hours	-	-
Equal opportunities/discrimination (w)	B2_management > B2_workers	1
Health and safety (w)	-	-
Social benefits/social security (w)	-	-
Employment relationship	B1_management < B1_workers; B3_management < B3_workers	2
Trading relationships	-	-
Next generation farmers	-	-
Equal opportunities/discrimination (f)	-	-
Social benefits/social security (f)	B2_management > B2_farmers; D2_management > D2_farmers	2
Health and safety (f)	-	-
Promoting social responsibility	-	-
Supplier relationships	-	-
Wealth distribution	-	-
Access to material resources	B2_management > B2_workers; D3_management > D3_farmers	2
Access to immaterial resources	D3_management > D3_farmers; D4_management < D4_workers	2
Cultural heritage	B2_management > B2_workers	1
Safe and healthy working conditions	D3_management > D3_workers; D3_management > D3_farmers; D4_management > D4_workers; D4_management > D4_farmers	4
Community engagement	D2_management > D2_workers; D2_management > D2_farmers	2
Local employment	-	-
Mean across all impact subcategories	-	-

S2 Contextual factors

S2.1 Forced labor

None of the farmers or plantations in our case studies appeared on the Brazilian Government's latest 'dirty list' of slave labor (Ministério do Trabalho e Emprego 2025). However, there are reported cases of slave labor in all municipalities where the case studies are located and Minas Gerais is the state in Brazil with most reported cases, particularly in the agricultural sector (Hjerl Hansen et al. 2016; Ministério do Trabalho e Emprego 2025). One of the interviewees, when talking about previous work experiences, said: *"The beds were made of grass. We didn't have running water, a filter to drink water, or a toilet. There was a toilet outside. And everyone slept together. If you were hungry, you stayed hungry because there was no stove to cook on. Or you'd buy a small pot to make coffee and eat cookies." (Rural/farm worker, B3)*

In the DRC, we encountered no reports of forced labor. However, the working conditions are at times very precarious and to the best of our knowledge, there is no literature on it. One coffee sorter (D3) said: *"We don't eat during the day, not even a sugar cane, you sometimes leave the house without eating anything, you spend the day without eating anything until 3pm when you come home again."*

S2.2 Sexual harassment

Sexual harassment is a widespread problem in coffee production, although it is not widely discussed (Sachs et al. 2019). Some scientific and grey literature exists for Brazil (Ergon and ALDI 2021; Raposeiras and Couto 2021) and in eastern DRC, there is an alarming high rate of sexual violence, however, mostly perpetrated by militant groups rather than actors in the coffee value chain (MSF 2025).

S2.3 Fair competition and respect of intellectual property rights

Research on geographical indications of origin indicates that only a very small number of coffee origins are protected, with many famous single-origin coffees being sold without legal protection, enabling free-riding and misleading origin claims that disadvantage actors in origin countries (Teuber 2007; Schüssler 2009). This can undermine fair competition and limit coffee producers' ability to capture the value created by reputation and quality differentiation.

S2.4 Delocalization and migration

In Brazil, most seasonal coffee workers in the South of Minas Gerais come from poorer regions in the north of the state, or from states north of Minas Gerais. These workers usually face worse working conditions than local seasonal workers do (Hjerl Hansen et al. 2016). In eastern DRC, the conflicts over the past 30 years have led to forced internal migration and ultra-precarious living conditions, leaving many people with no choices. Most metayers of case study D1 are such internal migrants.

S2.5 Respect of indigenous rights

In both countries, the respect of indigenous rights is not ensured. Indigenous communities in Brazil are particularly vulnerable to the expansion of the agribusiness (Ferrante et al. 2025), and in the DRC, the protection of indigenous peoples, paricularly Batwa groups remains weak (International Alert 2025). The Batwa indigenous peoples are present in both case study regions, but they were not involved in any of our case studies. Unfortunately, we could not find out whether this was by choice or due to systematic exclusion.

S2.6 Secure living conditions

Looking at the Global Peace Index 2024, which reflects safety and security in countries, both the DRC and Brazil rank towards the end of the list: the DRC is in 160th place out of 163, and Brazil in 130th place (Institute for Economics & Peace 2025). In eastern DRC, armed conflicts further escalated since January 2025. Previously, conflicts and instabilities since 1994 have heavily affected coffee production and led to a decline in production (Richey and Ponte 2021). Currently, many international coffee buyers dropped out, and capital is massively lacking, leaving many farmers and cooperatives with no other option than selling their coffee for very low prices to middlemen or smuggling it to Rwanda. Furthermore, many coffee farmers became refugees and had to abandon their fields.

S2.7 Structural discrimination

Structural discrimination against women is quite well-documented in the coffee value chain (Sigrist et al. 2025, Sigrist et al., under review). In Brazil and the DRC, women usually earn less in the same jobs, face barriers in accessing land and credit, and have little visibility and decision-making power, despite often performing most of the farm labor (Sachs et al. 2019; Ergon and ALDI 2021; Grant 2021; Franck and Prapha 2021; Sigrist et al. 2025). In Brazil, racial and intersectional discrimination are less documented or not documented at all in the coffee value chain, despite it being an important issue (Crenshaw 1994; Raposeiras and Couto 2021). People who identify as non-white make up more than half of rural/farm workers. However, they rarely own (large-scale) farms or run cooperatives driving the Brazilian coffee sector (Belandi 2019). In our overall sample, slightly more men than women earned a salary above the minimum wage, and in Brazil, white workers were more likely than non-white workers to earn above the minimum wage, reflecting existing structural inequalities. (see Table SI 1). For ethnic discrimination in the DRC, see S2.5 Respect of indigenous rights.

S3 Figures

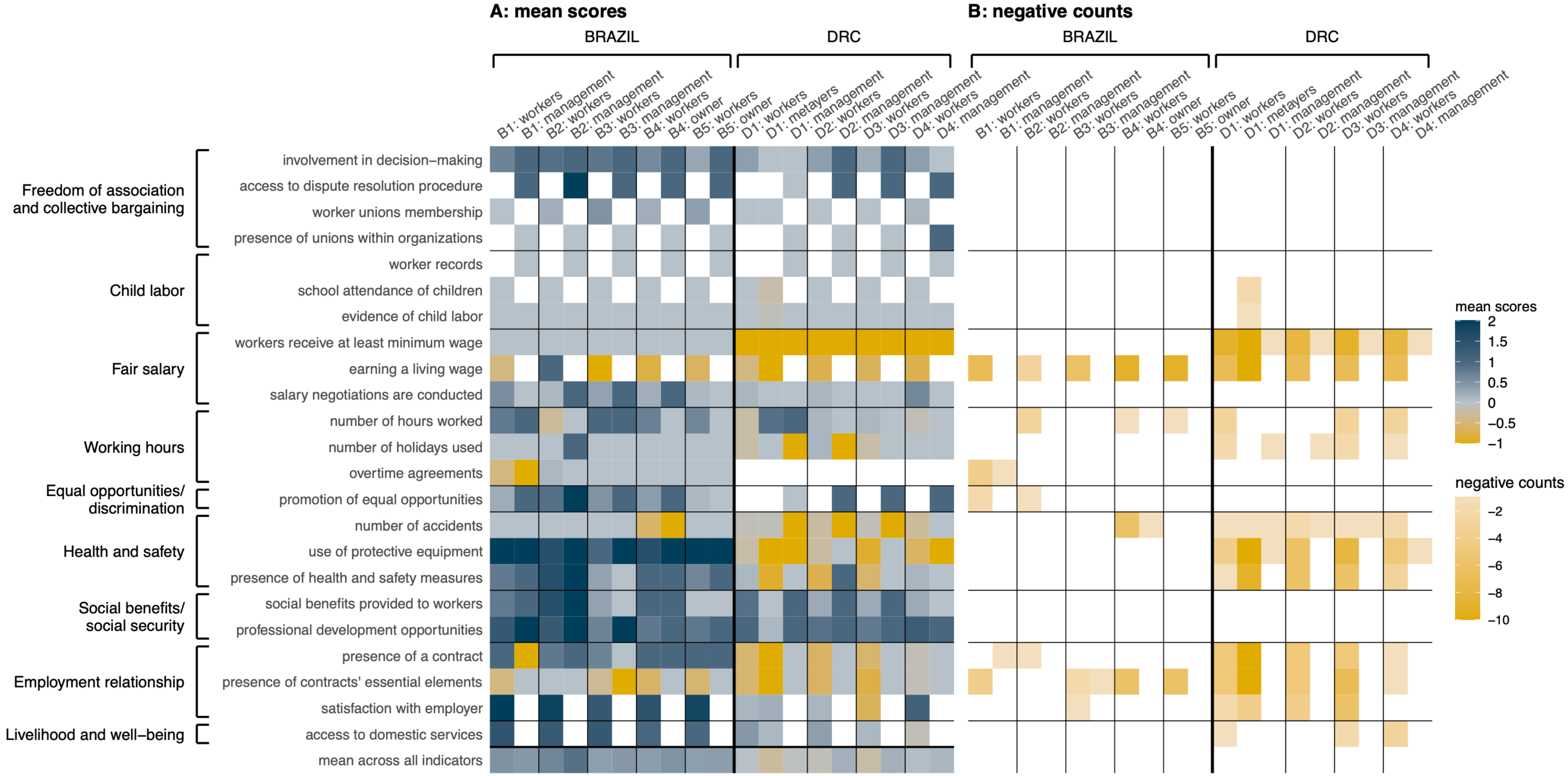

Figure SI 1: The heatmaps show the results of the social performance of the different case studies divided by group of interviewee (workers, metayers, farmers, management). The results are displayed for each indicator grouped by the corresponding impact subcategory for the stakeholder category worker. The x-axis shows the country, case study, and group of interviewee, and the y-axis shows the indicator and impact subcategory. Heatmap A shows the mean performance scores (on the -1 to 2 reference scale) for each group of interviewee, while the heatmap B shows the total number of individual responses scored at level -1 (negative counts, i.e. non-compliant cases) for each interviewee group. The negative counts are displayed as negative values for visual distinction; the values represent response counts, not performance scores. For more information on the scoring method, see section 2.3.1 of the main text.

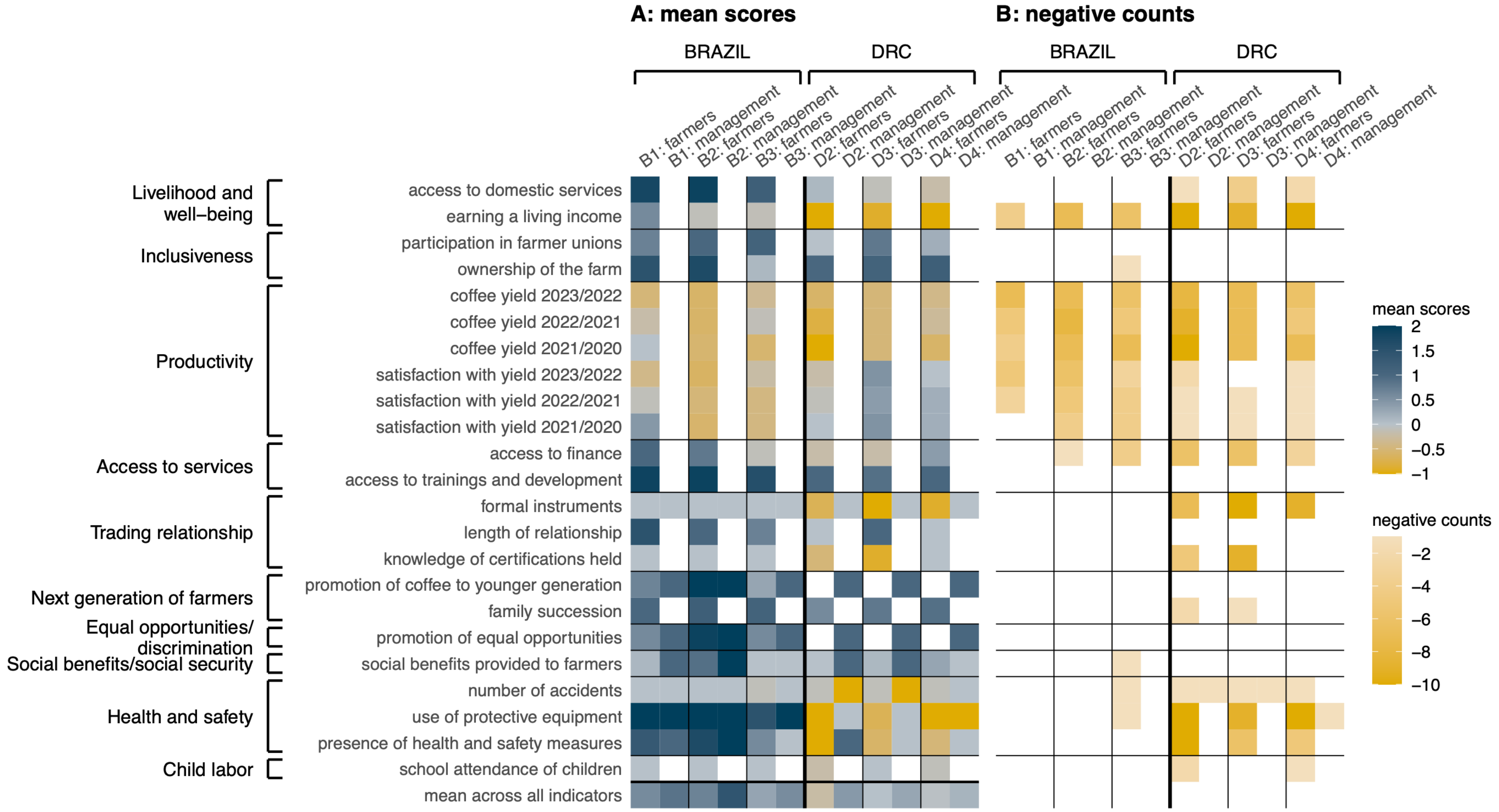

Figure SI 2: The heatmaps show the results of the social performance of the different case studies divided by group of interviewee (workers, metayers, farmers, management). The results are displayed for each indicator grouped by the corresponding impact subcategory for the stakeholder category farmer. The x-axis shows the country, case study, and group of interviewee, and the y-axis shows the indicator and impact subcategory. Heatmap A shows the mean performance scores (on the -1 to 2 reference scale) for each group of interviewee, while the heatmap B shows total number of individual responses scored at level -1 (negative counts, i.e. non-compliant cases) for each interviewee group. The negative counts are displayed as negative values for visual distinction; the values represent response counts, not performance scores. For more information on the scoring method, see section 2.3.1 of the main text.

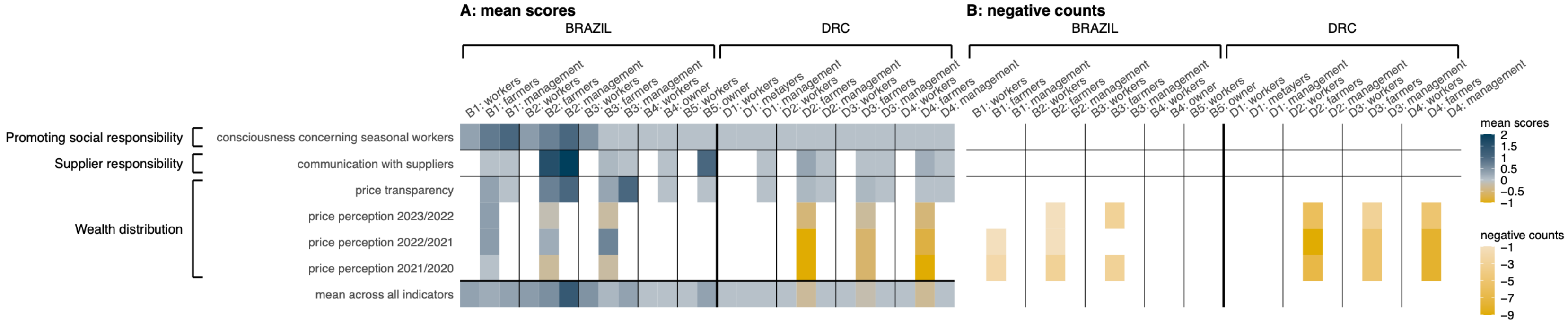

Figure SI 3: The heatmaps show the results of the social performance of the different case studies divided by group of interviewee (workers, metayers, farmers, management). The results are displayed for each indicator grouped by the corresponding impact subcategory for the stakeholder category value chain actors. The x-axis shows the country, case study, and group of interviewee, and the y-axis shows the indicator and impact subcategory. Heatmap A shows the mean performance scores (on the -1 to 2 reference scale) for each group of interviewee, while the heatmap B shows the total number of individual responses scored at level -1 (negative counts, i.e. non-compliant cases) for each interviewee group. The negative counts are displayed as negative values for visual distinction; the values represent response counts, not performance scores. For more information on the scoring method, see section 2.3.1 of the main text.

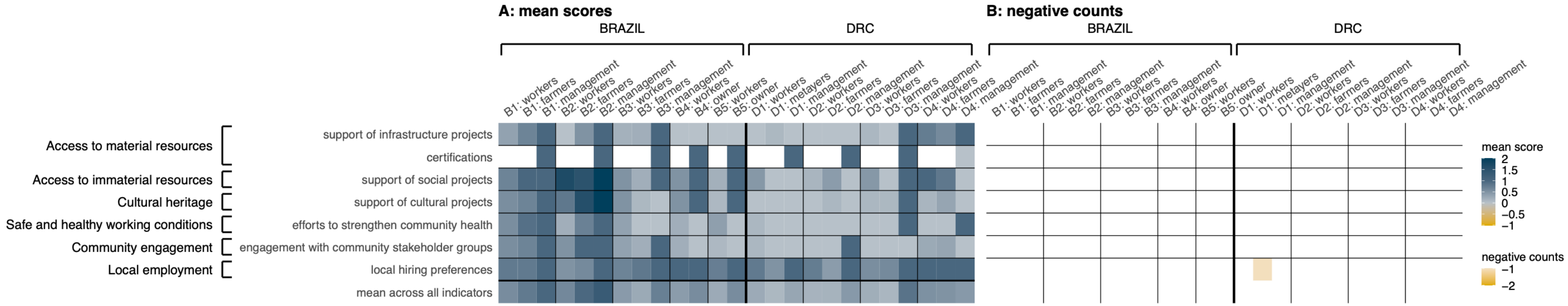

Figure SI 4: The heatmaps show the results of the social performance of the different case studies divided by group of interviewee (workers, metayers, farmers, management). The results are displayed for each indicator grouped by the corresponding impact subcategory for the stakeholder category local community. The x-axis shows the country, case study, and group of interviewee, and the y-axis shows the indicator and impact subcategory. Heatmap A shows the mean performance scores (on the -1 to 2 reference scale) for each group of interviewee, while the heatmap B shows the total number of individual responses scored at level -1 (negative counts, i.e. non-compliant cases) for each interviewee group. The negative counts are displayed as negative values for visual distinction; the values represent response counts, not performance scores. For more information on the scoring method, see section 2.3.1 of the main text.

S4 References

Belandi C (2019) Blacks and browns are the minority heading large agricultural establishments | News Agency. In: Agência de Notícias - IBGE. https://agenciadenoticias.ibge.gov.br/en/agencia-news/2184-news-agency/news/26175-blacks-and-browns-are-the-minority-heading-large-agricultural-establishments. Accessed 2 Feb 2026

Crenshaw KW (1994) Mapping the Margins: Intersectionality, Identity Politics, and Violence Against Women of Color. In: The Public Nature of Private Violence, 1st Edition. Routledge, pp 93–118

Ergon and ALDI (2021) HUMAN RIGHTS IMPACT ASSESSMENT REPORT, COFFEE FROM BRAZIL. ALDI SOUTH Group

Ferrante L, Vilani RM, Apurinã W, et al (2025) Brazil's Indigenous lands face legal setbacks. Science 389:465–465. https://doi.org/10.1126/science.ady1746

Franck A, Prapha A (2021) Not in This Together: How supermarkets became pandemic winners while women workers are losing out. Oxfam

Grant T (2021) Gender equity in the Democratic Republic of Congo's coffee sector. In: Perfect Daily Grind. https://perfectdailygrind.com/2021/04/gender-equity-in-the-democratic-republic-of-congos-coffee-sector/. Accessed 2 Feb 2026

Hjerl Hansen J, Christensen AB, Andersen Thing D, Sjöström T (2016) BITTER Coffee, Slavery-like Working Conditions and deadly Pesticides on Brazilian Coffee Plantations. DANWATCH

Institute for Economics & Peace (2025) Global Peace Index 2025: Identifying and Measuring the Factors that Drive Peace. Sydney

International Alert (2025) Protecting Indigenous peoples' rights and nature in eastern DRC: the role of peacebuilding. https://www.international-alert.org/stories/protecting-indigenous-peoples-rights-and-nature-in-eastern-drc/. Accessed 2 Feb 2026

Ministério do Trabalho e Emprego (2025) Cadastro de Empregadores que tenham submetido trabalhadores a condições análogas à de escravo, Portaria Interministerial MTE/MDHC/MIR nº 18 de 13/09/2024. Brazil

MSF (2025) Alarming levels of sexual violence in eastern DR Congo. https://www.doctorswithoutborders.org/latest/alarming-levels-sexual-violence-eastern-drc. Accessed 2 Feb 2026

Raposeiras I, Couto C (2021) O livro da barista - instrumentos teóricos para um bom profissional do café. Coffee Lab, São Paulo

Richey LA, Ponte S (2021) Brand Aid and coffee value chain development interventions: Is Starbucks working aid out of business? World Dev 143:105193. https://doi.org/10.1016/j.worlddev.2020.105193

Sachs J, Cordes KY, Rising J, et al (2019) Ensuring Economic Viability and Sustainability of Coffee Production. Columbia Center on Sustainable Investment

Schüssler L (2009) Protecting 'Single-Origin Coffee' within the Global Coffee Market: The Role of Geographical Indications and Trademarks. Estey Centre Journal of International Law and Trade Policy 10:149–185

Sigrist M, Schneider G, Thom B, Jacobi J (under review) Power asymmetries in the coffee value chain: Strategies and resistance at the margins in Minas Gerais, Brazil. The Journal of Peasant Studies

Sigrist M, Thom B, Gabriela S, et al (2025) Coffee Work Outside Plantations - Emancipation, Fame and Limits for the Next Generation of Coffee Growers in Minas Gerais (Brazil). Anthropol Soc 49:193–216. https://doi.org/https://doi.org/10.7202/1119573ar

Teuber R (2007) Geographical Indications of Origin as a Tool of Product Differentiation: The Case of Coffee, Discussion Paper No. 33. Justus-Liebig-Universität Gießen, Zentrum für Internationale Entwicklungs- und Umweltforschung (ZEU), Giessen
