# Supplementary material for: Towards greater sensitivity: advancing social life cycle assessment for the coffee sector in Brazil and the Democratic Republic of the Congo
Source: Int J Life Cycle Assess. 2026 Oct 18;31(11):190. doi: 10.1007/s11367-026-02760-8 (PMC13677624; doi:10.1007/s11367-026-02760-8)
Supplement: Supplementary file 2 [file 11367_2026_2760_MOESM3_ESM.pdf]

Online Resource 3

Towards greater sensitivity: advancing social life cycle assessment for the coffee sector in Brazil and the Democratic Republic of the Congo

The International Journal of Life Cycle Assessment

Authors: Braida Thom[1], Lea Fabritius[1], Marie Sigrist[1], Didace Banyanga[2], Stephan Pfister[3], Réussite Bugale Malembaka[1], Gabriela Schneider[4], Kokou Kintche[2], Johanna Jacobi[1]

[1]Agroecological Transitions, Department of Environmental Systems Sciences, ETH Zurich, Switzerland, Universitätstrasse 2, 8092 Zurich, Switzerland
[2] International Institute of Tropical Agriculture (IITA), Route Kavumu, Km 18, Bifurcation Birava, Site Kalambo, Bukavu, Democratic Republic Congo
[3] Ecological Systems Design, Department of Civil, Environmental and Geomatic Engineering, ETH Zurich, Switzerland, Laura-Hezner-Weg 7, 8093 Zurich, Switzerland
[4] Programa de Ciencias Sociais em Desenvolvimento, Agricultura e Sociedade, Universidade Federal Rural do Rio de Janeiro (UFRRJ), BR-465, Km 7 - Seropédica, Rio de Janeiro, Brazil

Correspoding author: Braida Thom (braida.thom@gmail.com)

Producer_ID:

Inventário social: Produtores/as de café

****Antes de iniciar a entrevista, mostraremos a você o formulário de informações, contato e consentimento****

Para todas as perguntas a seguir: Por favor nos informar caso não souber a resposta. Tenha em mente que você sempre tem a opção de não responder a uma pergunta, sem fornecer um motivo.

Você é membro de uma cooperativa de café? ☐ sim ☐ não [coop]

Se sim, qual cooperativa e desde quando?

________ desde quando? ________ [coop_1] [coop_1_s]

________ desde quando? ________ [coop_2] [coop_2_s]

________ desde quando? ________ [coop_3] [coop_3_s]

Se sim, qual é a taxa de associação da cooperativa? [fee]

Você é membro de uma organização, associação ou sindicato de agricultores? [org] ☐ sim ☐ não

Se sim, qual associação ou sindicato e desde quando?

________ desde quando? ________ [org_1] [org_1_s]

________ desde quando? ________ [org_2] [org_2_s]

________ desde quando? ________ [org_3] [org_3_s]

Se sim, qual é o objetivo da associação ou união? [org_o]

Tamanho total da fazenda/do sítio (terras cultivadas e improdutivas; terras alugadas e próprias):

________ *ha* [f_size]

Área alocada para o café:

________ *ha* [c_size]

Área de café produtivo (árvores que produzem cerejas):

________ *ha* [p_size]

Área com vegetação natural nativa (mata/capoeira):

________ *ha* [n_size]

Área com reflorestamento (mata/capoeira):

________ *ha* [r_size]

Número de lavouras de café: ________ [n_farms]

Idade dessas lavouras de café:

________ *anos* [age_f1]

________ *anos* [age_f2]

________ *anos* [age_f3]

________ *anos* [age_f4]

Número de cafeeiros no total ou por hectare: ________ [n_ctree]

Que variedades de café você cultiva? [var]

Qual foi a produção total de café nos últimos três anos? (kg de café descascado/bica corrido, grãos crus, kg de cerejas, kg/ha, sacas: **se indicado em sacas, quantos kg contém uma saca)**

Colheita **2023** : ____________________ [yield_23]

Colheita **2022** : ____________________ [yield_22]

Colheita **2021** : ____________________ [yield_21]

Qual é a colheita normal de café em um ano ruim? ____________________ [good_yield]

Qual é a colheita normal de café em um ano bom? ____________________ [bad_yield]

Que outras culturas e hortaliças você produz em sua fazenda / seu sítio (culturas de subsistência e comerciais)?

Cultura: __________ [crop1] Colheita 2022 ou 2023: __________ [y_c1_22] Como ela é cultivada? [c1_m]
- ☐ Consorciada com café
- ☐ Monocultura
- ☐ Consorciada com outras culturas
- ☐ Outro

Cultura: __________ [crop2] Colheita 2022 ou 2023: __________ [y_c2_22] Como ela é cultivada? [c2_m]
- ☐ Consorciada com café
- ☐ Monocultura
- ☐ Consorciada com outras culturas
- ☐ Outro

Cultura: __________ [crop3] Colheita 2022 ou 2023: __________ [y_c3_22] Como ela é cultivada? [c3_m]
- ☐ Consorciada com café
- ☐ Monocultura
- ☐ Consorciada com outras culturas
- ☐ Outro

Cultura: __________ [crop4] Colheita 2022 ou 2023: __________ [y_c4_22] Como ela é cultivada? [c4_m]
- ☐ Consorciada com café
- ☐ Monocultura
- ☐ Consorciada com outras culturas
- ☐ Outro

Cultura: __________ [crop5] Colheita 2022 ou 2023: __________ [y_c5_22] Como ela é cultivada? [c5_m]
- ☐ Consorciada com café
- ☐ Monocultura
- ☐ Consorciada com outras culturas
- ☐ Outro

Cultura: __________ [crop6] Colheita 2022 ou 2023: __________ [y_c6_22] Como ela é cultivada? [c6_m]
- ☐ Consorciada com café
- ☐ Monocultura
- ☐ Consorciada com outras culturas
- ☐ Outro

Venda

Qual foi o preço que você recebeu pelo seu café nos últimos três anos? (total, preço por unidade de cerejas, por unidade de café descascado/bica corrido, por unidade de café cru: **se for dado em sacas, quantos kg contém uma saca)**

Preço **2023**: ____________________ [price_23]

Preço **2022**: ____________________ [price_22]

Preço **2021**: ____________________ [price_21]

O que você considera um bom preço para o café? ______________________________ [price_good]

O que você considera um preço ruim para o café? ______________________________ [price_bad]

Para quem você vende seu café? [buy]

Quais prêmios você recebeu nos últimos três anos (total, prêmios por unidade de cerejas, por unidade de café descascado/bica corrido, por unidade de café cru)?

Prêmios **2023**: ______________________________ [prim_23]

Prêmios **2022**: ______________________________ [prim_22]

Prêmios **2021** : ______________________________ [prim_21]

Por que e de quem você recebeu os prêmios? [prim_why]

Qual foi a sua renda total em **2022** (atividades agrícolas e não agrícolas, remessas de familiares)?

__________ R$/ano de produção de café ☐ Não quero responder a essa pergunta [inc_c]

__________ R$/ano de __________________ [inc_o1] [act_1]

__________ R$/ano de __________________ [inc_o2] [act_2]

__________ R$/ano de __________________ [inc_o3] [act_3]

__________ R$/ano de __________________ [inc_o4] [act_4]

Informações sobre o sistema de gestão

Qual é o seu sistema de gestão de café *(várias respostas possíveis)?* [manag]

Certificação

- ☐ Certifica Minas
- ☐ Rainforest Alliance
- ☐ 4C
- ☐ Certificação Fairtrade
- ☐ Certificação orgânica (p. ex., orgânico da UE): ____________________ [manag_o]

- ☐ Outras certificações: ____________________ [manag_oc]

- ☐ Orgânico em transição

Área certificada e em transição:

____________________ [manag_s]

- ☐ Denominação de origem protegida: ____________________ [manag_dop]
- ☐ Indicação geográfica protegida: ____________________ [manag_igp]

- ☐ Convencional
- ☐ Orgânico não certificado
- ☐ Sem uso de agrotóxicos, mas com uso de adubos minerais
- ☐ Sem uso de adubos minerais, mas com uso de agrotóxicos

- ☐ Monocultura
- ☐ Cultivo consorciado com culturas comestíveis
- ☐ Cultivo consorciado com culturas de cobertura no meio das fileiras de café
- ☐ Com algumas árvores de sombra
- ☐ Agrofloresta (policultura)
- ☐ Outro sistema: ____________________ [manag_os]

Parte social

A moradia atual da família é?

- ☐ Própria ou já paga [house_t]
- ☐ Alugada
- ☐ Paga prestação
- ☐ Cedida
- ☐ Outra: [house_t_o]

A sua casa é feita de:

- ☐ Alvenaria [house_m]
- ☐ Madeira
- ☐ Taipa não revestida (ou pau a pique)
- ☐ Outros: [house_m_ol

Quantos cômodos tem a sua casa? (contar todos, exceto o banheiro e a cozinha) [room]

Sua casa tem:

- ☐ Água encanada
- ☐ Banheiro dentro de casa
- ☐ Fossa séptica
- ☐ Fossa comum (rudimentar)
- ☐ Não tem instalação sanitária
- ☐ Outro: ______________________
- ☐ Rede geral

Qual é a fonte de energia?

- ☐ Elétrica [cook]
- ☐ Gás
- ☐ Querosene
- ☐ Gerador
- ☐ Outro: ______________________ [cook_o]

Quais os principais utensílios domésticos existentes na moradia?

- ☐ Fogão a lenha [asset1]
- ☐ Fogão a gás [asset2]
- ☐ Geladeira [asset3]
- ☐ Televisor [asset4]
- ☐ Rádio [asset5]
- ☐ Freezer [asset6]
- ☐ Antena Parabólica [asset7]
- ☐ Computador [asset8]
- ☐ Máquina de lavar roupa [asset9]
- ☐ Telefone [asset10]

Quais desses bens você ou algum membro de sua família tem?

- ☐ Automóvel [asset13]
- ☐ Moto [asset14]
- ☐ Trator [asset15]
- ☐ Caminhão [asset16]

Você tem acesso a crédito acessível ou pré-financiamento para insumos? ☐ sim ☐ não [credit]

Se sim, por quem? [credit_p]

☐ Pronaf: Custeio (insumos) ☐ Pronaf: Investimento ☐ Outro: ______________________ [credit_po]

Você tem acesso a formação em boas práticas agrícolas/serviços de extensão? ☐ sim ☐ não [gap_tr]

Se sim, sobre quais temas/quais tipos de formações/serviços de extensão? [gap_tr_t]

Se sim, quem fornece essas formações? ☐ Cooperativa: ____________________ [gap_tr_p]

☐ Fornecedor de insumos ☐ EMATER ☐ Estado ☐ ONGs ☐ Outro: ____________________ [gap_tr_n] [gap_tr_po]

Saúde e seguridade social

Você tem um seguro/plano de saúde? ☐ sim ☐ não [h_insu]

Você recebe algums benefícios sociais / previdência social (p.ex., aposentadoria, créditos)? ☐ sim ☐ não [sobe]

Se sim, que tipo de benefícios sociais você recebe? [sobe_t]

Se sim, de quem você recebe esses benefícios? ☐ Cooperativa: ____________________ [sobe_p]

☐ Fornecedor de insumos ☐ EMATER ☐ Estado ☐ ONGs ☐ Outro: ____________________ [sobe_n] [sobe_po]

Se você, seus familiares ou seus/suas empregados/as estiverem realizando atividades perigosas (p.ex, aplicação de agrotóxicos), alguém está usando equipamento de proteção? [pr]

☐ Todos ☐ Ninguém ☐ Outro: ____ [pr_o]

No ano passado, teve algum acidente em sua fazenda / seu sítio no qual você, um membro de sua família ou um/a empregado/a se feriu? ☐ sim ☐ não [acc]

Se sim, que acidente ocorreu e quem ficou ferido? [acc_t]

O que você está fazendo para evitar acidentes em sua fazenda / seu sítio? [acc_pr]

Você tem acesso a formações sobre riscos a saúde e a prevenção de doenças? ☐ sim ☐ não [h_tr]

Se sim, sobre quais temas/quais tipos de formações? [h_tr_t]

Se sim, quem fornece essas formações? ☐ Cooperativa: ____________________ [h_tr_p]

☐ Fornecedor de insumos ☐ EMATER ☐ Estado ☐ ONGs ☐ Outro: ____________________ [h_tr_n] [h_tr_po]

Trabalhadores/as familiares

Quem da família trabalha com você na lavoura? [employf]

☐ Filhos ☐ Esposo/a ☐ Outros membros da família ☐ Trabalha sozinho/a

Você paga o trabalho deles? [employf_sa]

☐ sim ☐ não

Empregados/as ou trabalhadores/as

Você tem empregados/as ou trabalhadores/as remunerados em sua fazenda / seu sítio? ☐ sim ☐ não [employ]

Se sim, complete a tabela abaixo (uma linha por pessoa!) e responda às perguntas da próxima página.

**Atividade/função** (**#diaristas**) [employ_f]	**Status de emprego** (diarista, sazonal/safreiro/safrista, permanente, **#dias**) [employ_s]	**Idade das pessoas** [employ_a]	**Gênero** [employ_g]	**Em que estado a pessoa nasceu e que e sua identificação étnico-racial?** [e_state] [employ_e]	**Salário** (por hora, dia ou mês) [employ_sa]

**Atividade/função** (**#diaristas**) [employ_f]	**Status de emprego** (diarista, sazonal/safreiro/safrista, permanente, **#dias**) [employ_s]	**Idade das pessoas** [employ_a]	**Gênero** [employ_g]	**Em que estado a pessoa nasceu e que e sua identificação étnico-racial?** [e_state] [employ_e]	**Salário** (por hora, dia ou mês) [employ_sa]

Quantas horas por dia eles/as trabalham e quanto tempo duram os intervalos? [work_h]

Para empregados/as, trabalhadores/as sazonais e permanentes, quantos dias por semana eles/as trabalham? [work_d]

Para empregados/as, trabalhadores/as permanentes, quantos dias de férias remuneradas eles/as têm por ano (em dias)? [vac_d]

Há algum limite de idade para as pessoas que você emprega? ☐ sim ☐ não

Se sim, qual é o limite? [work_al]

[work_al_t]

Por favor, responda às próximas perguntas sobre seus empregados/as, trabalhadores/as com sim ou não. Se a resposta for sim, especifique se ela se aplica a todos os funcionários ou apenas a alguns.

Você lhes dá um contrato de trabalho?	☐ não, nenhum	☐ sim, todos	☐ sim, alguns: ____	[work_c] [work_ct]
Eles têm uma carteira de trabalho?	☐ não, nenhum	☐ sim, todos	☐ sim, alguns: ____	[w_card] [w_cardt]
Vôce concede a eles um décimo terceiro salário?	☐ não, nenhum	☐ sim, todos	☐ sim, alguns: ____	[13_w] [13_wt]
Você paga horas extras ou as concede como férias remuneradas?	☐ não, nenhum	☐ sim, todos	☐ sim, alguns: ____	[overt_w] [overt_wt]
Se eles trabalharem até tarde ou durante a noite, você lhes dá um adicional noturno?	☐ não, nenhum	☐ sim, todos	☐ sim, alguns: ____	[night_w] [night_wt]
Se eles realizarem atividades insalubres ou perigosas, você lhes dá um adicional de insalubres ou periculosidade?	☐ não, nenhum	☐ sim, todos	☐ sim, alguns: ____	[add_w] [add_wt]
Você fornece a eles equipamentos de proteção?	☐ não, nenhum	☐ sim, todos	☐ sim, alguns: ____	[pr_w] [pr_wt]
Eles têm um seguro/plano de saúde?	☐ não, nenhum	☐ sim, todos	☐ sim, alguns: ____	[h_insu_w] [h_insu_wt]
Se eles tiverem um bebê, eles recebem licença-paternidade?	☐ não, nenhum	☐ sim, todos	☐ sim, alguns: ____	[pleave_w] [pleave_wt]
Você faz depósitos de FGTS (Fundo de Garantia do Tempo de Serviço) para eles?	☐ não, nenhum	☐ sim, todos	☐ sim, alguns: ____	[fgts_w] [fgts_wt]
Eles têm um seguro-desemprego?	☐ não, nenhum	☐ sim, todos	☐ sim, alguns: ____	[unemp_w] [unemp_wt]
Você concede previdência social, incluindo auxílio-doença e aposentadoria ou recolhe contribuições previdenciárias?	☐ não, nenhum	☐ sim, todos	☐ sim, alguns: ____	[sobe_w] [sobe_wt]
Você concede outros benefícios sociais?	☐ não, nenhum	☐ sim, todos	☐ sim, alguns: ____	[sobeo_w] [sobeo_wt]

Se sim, que tipo de benefícios sociais? [sobeo_w_t]

Você dá a eles/as formações sobre riscos à saúde e prevenção de doenças (ou outros temas)? [h_tr_w] [h_tr_wt]

☐ não, nenhum ☐ sim, todos ☐ sim, alguns: ______________

Se sim, sobre quais temas / quais tipos de formações ? [h_tr_wt_t]

Você dá preferência à contratação de empregados/as ou trabalhadores/as locais? ☐ sim ☐ não [lh]

Se sim, como você faz isso? [lh_t]

Você fornece moradia ou alimentação para seus empregados/as ou trabalhadores/as? ☐ sim ☐ não [hf]

Se sim, o que há na moradia?
- ☐ Camas [hf1]
- ☐ Cozinha [hf2]
- ☐ Equipamentos de cozinha [hf3]
- ☐ Geladeira [hf4]
- ☐ Banheiro [hf5]
- ☐ Ar-conditionado ou ventilador [hf6]

Se sim, quantas pessoas compartilham um quarto? ______________ [hf_p]

Se sim, quanto custa a moradia? ______________ [hf_ch]

Se sim, quanto custa a alimentação? ______________ [hf_cf]

Colaboração com a cooperativa/empresa

Você tem um contrato com sua cooperativa (p.ex., uma garantia de que eles compram o café)? ☐ sim ☐ não [contr]

Se sim, qual é o tipo de contrato? [contr_t]

Você está envolvido/a nos processos de tomada de decisões da cooperativa? ☐ sim ☐ não [deci]

Se sim, como? [deci_i]

Você está envolvido/a na definição dos preços do café da cooperativa? ☐ sim ☐ não [nego]

Se sim, como? [nego_i]

Há alguma medida para promover a diversidade de gênero na cooperativa? ☐ sim ☐ não [equ_opp]

Se sim, que tipo de medidas?

A cooperativa da qual você é membro está fazendo alguma coisa para promover o cultivo de café entre a geração mais jovem? ☐ sim ☐ não [p_succ]

Se sim, o que exatamente? [p_succ_t]

A cooperativa da qual você é membro implementou algum projeto social (p.ex., apoio a escolas locais) ao qual os cooperados/as e/ou a comunidade local (além dos cooperados/as) têm acesso? ☐ sim ☐ não [soc_pr]

Se sim, que tipos de projetos sociais e para quem? [soc_pr_t]

A cooperativa da qual você é membro implementou algum projeto de infraestrutura (p.ex., construção de estradas) ao qual os cooperados/as e/ou a comunidade local (além dos cooperados/as) têm acesso? ☐ sim ☐ não [inf_pr]

Se sim, que tipos de projetos de infraestrutura e para quem? [inf_pr_t]

A cooperativa da qual você é membro implementou algum projeto para apoiar a integração de trabalhadores migrantes na comunidade? ☐ sim ☐ não [int_pr]

Se sim, que tipo de projetos? [int_pr_t]

A cooperativa da qual você é membro implementou algum projeto para apoiar ou promover o patrimônio cultural da região (p.ex., financiando atividades culturais ou expressando o patrimônio cultural em produtos)? ☐ sim ☐ não [cult_pr]

Se sim, que tipo de projetos? [cult_pr_t]

A cooperativa da qual você é membro ofereceu formações sobre riscos à saúde e prevenção de doenças para a comunidade local (além dos cooperados/as)? ☐ sim ☐ não [h_tr_c]

Se sim, sobre quais temas / quais tipos de formações? [h_tr_ct]

A cooperativa da qual você é membro implementou algum projeto para proteger os direitos dos membros de comunidades indígenas? ☐ sim ☐ não [ind_pr]

Se sim, que tipo de projetos? [ind_pr_t]

A cooperativa da qual você é membro trabalha com grupos comunitários (p.ex., a igreja)? ☐ sim ☐ não [collab]

Se sim, com quais tipos de grupos comunitários e para quais projetos? [collab_t]

A cooperativa da qual você é membro dá ênfase à contratação de empregados/as locais? ☐ sim ☐ não [lh_c]

Se sim, como eles/as estão implementando isso? [lh_c_t]

Sucessão

Você quer que seus/suas filhos/as continuem cultivando café? Por que você quer ou não? [succ]

Quais são seus desejos para o futuro? [future]

Idade: ______________________ [age]

Gênero: ☐ m ☐ f [sex]
☐ outro: __________

Em que estado você nasceu? ______________ [state]

Dentre as opções branco, preto, pardo, amarelo e indígena, com qual delas você mais se identifica? [ethni]

☐ branco ☐ preto ☐ pardo ☐ amarelo ☐ indígena

Educação: [edu]

☐ analfabeto / fundamental I (primário) incompleto
☐ fundamental I completo / fundamental II incompleto
☐ fundamental II completo (ginásio) / médio incompleto
☐ médio completo (colegial) / superior incompleto
☐ superior completo

Propriedade da terra: [entit]

☐ própria com título
☐ própria sem título
☐ terras arrendadas
sistema de pagamento
☐ meeiro ☐ terceiro ☐ quartista
☐ pagamento em dinheiro: ______________ *R$/ano*
☐ outro: [entit_o]

Tamanho da família: ______________ [hh_size]

Quantas crianças em sua casa estão em idade escolar? #meninas: ______ [f_kids]
#meninos: ______ [m_kids]

Quantos vão à escola: #meninas: ______ [f_scho]
#meninos: ______ [m_scho]

O que fazem as crianças em idade escolar que não frequentam a escola? [scho_a]

Entre as crianças em idade escolar que não vão à escola, por que elas não vão? [scho_w]
